# (−)-Gallocatechin gallate from green tea rescues cognitive impairment through restoring hippocampal silent synapses in post-menopausal depression

**DOI:** 10.1038/s41598-020-79287-x

**Published:** 2021-01-13

**Authors:** Sukjin Ko, Won Seuk Jang, Ji-Hyun Jeong, Ji Woong Ahn, Young-Hwan Kim, Sohyun Kim, Hyeon Kyeong Chae, Seungsoo Chung

**Affiliations:** 1 Brain Korea 21 Plus Project for Medical Science, Department of Physiology, Yonsei University College of Medicine, Seoul, 03722 Republic of Korea; 2 Department of Medical Engineering, Yonsei University College of Medicine, Seoul, 03722 Republic of Korea; 3 BnH Research Co., LTD., Goyang-si, Gyeonggi-do 10594 Republic of Korea

**Keywords:** Neuroscience, Physiology, Neurology

## Abstract

Post-menopausal depression (PMD) is a common psychological disorder accompanied by a cognitive deficit, which is caused by a series of uncontrolled emotional disruptions by strong environmental stressors during menopause. To overcome PMD-induced cognitive deficit, Green tea has been suggested as a dietary supplement because of its ameliorating effect on cognitive dysfunction induced by normal aging or neurodegenerative syndromes; however, its clinical use to improve PMD-accompanied cognitive deficit is still limited due to the controversy for the active ingredients and ambiguous mechanism of its action. Here, we developed modified high-temperature-processed green tea extract (HTP-GTE), which showed lower neuronal toxicity than the conventional green tea extract (GTE). We also demonstrated that HTP-GTE administration prevented the development of learned helplessness (LH) in a rat post-menopausal model. Additionally, HTP-GTE improved LH-induced cognitive impairments simultaneously with rescued the long-term synaptic plasticity. This occurred via the restoration of silent synapse formation by increasing the hippocampal BDNF-tyrosine receptor kinase B pathway in the helpless ovariectomized (OVX) rats. Likewise, we also identified that (−)-gallocatechin gallate was the main contributor of the HTP-GTE effect. Our findings suggested that HTP-GTE has a potential as a preventive nutritional supplement to ameliorate cognitive dysfunctions associated with PMD.

## Introduction

Depressive disorder is a common but serious condition with severe symptoms such as uncontrolled emotional disruptions and cognitive dysfunction. Depression occurs in women twice as often as in men^1^ especially during menopause with loss of ovarian hormonal functions^2–4^. Depression is often caused after a series of uncontrolled emotional disruptions triggered by strong environmental stressors when the individuals are in clinically vulnerable conditions such as unbalanced hormonal state. In this case, we refer this specific depression as post-menopausal depression (PMD).

PMD plays a critical role in the alteration of synaptic efficacy of neuronal circuits in various brain regions including the cortex, hippocampus, and amygdala^5–7^. Of note, the disruption of hippocampal synaptic function, which under healthy conditions contributes to explicit memory and learning^8,9^, may result in cognitive impairments. Hippocampal volume and neurogenesis have been reported to be reduced in patients with major depressive disorder^10–13^. In addition, stress induces a decrease in spine density, and attenuates synaptic strength by both suppressing long-term potentiation (LTP) and by augmenting long-term depression (LTD) in the hippocampal circuit. This results in impairments of learning and memory, which has been demonstrated to increase the risk of depressive and apathetic behavior in rodents^14–17^. Taken together, this strongly suggests that the restoration of synaptic function in impaired hippocampal circuits is a potential therapeutic strategy for improving depression-induced learning and memory deficits.

Green tea (*Camellia sinensis*) is a widely consumed beverage, especially in Asia. It has been reported to have therapeutic effects such as amelioration of cognitive dysfunction induced by normal aging or neurodegenerative syndromes^18,19^. Akin to these data, green tea consumption could be a therapeutic solution for cognitive declines in psychopathological conditions, including post-menopausal depressive disorders. The main constituents are reported to be catechins and theanine^20^, responsible for the bioactive functions of green tea. Among them, (−)-epigallocathechin-3-gallate (EGCG) is a major catechin^21,22^ while theanine is a free amino acid present in high levels in green tea^23^. The studies on the effects of green tea on cognitive function are not always consistent because the concentrations of green tea and its constituents vary in each experiment depending on the manufacturing process. In fact, catechin content in canned green tea is relatively low because of the conversion of green tea catechins to their epimers (e.g. from EGCG to (−)-gallocatechin gallate (GCG)) during the heating processes of brewing and sterilization^24,25^. GCG has been reported to be more stable^26^ and bioactive than EGCG^27–29^ despite its small portion in the composition of unprocessed green tea. In addition, when green tea is administered as an extract with high doses of its bioactive ingredients, EGCG has been reported to be associated with other side effects, including hepatotoxicity and abnormal thyroid enlargement^5,30–33^. However, other isomers of green tea catechins, such as (−)-catechin (C), (−)-gallocatechin (GC), and GCG have yet been explored to test their functions in improving the impaired cognitive functions.

Nonetheless, the clinical use of green tea, especially as a dietary supplement are still limited due to the unknown mechanisms directly correlated to the cognitive functions. Thus, there is a need to develop a safe green tea derivative, which confers potent cognitive improvement without causing adverse side effects such as hepatotoxicity, even at high concentrations. Furthermore, detailed studies should be performed on potential derivatives to elucidate the signaling mechanisms of active ingredients responsible for improving cognitive impairments in depression.

We developed (−)-gallocatechin gallate (GCG)-enriched green tea extract, a modified green tea derivative in which GCG, an epimer of EGCG, was enriched by epimerization via heating and pH adjustment^24^. In addition, we used the learned helplessness (LH) model of depression to test the effects of HTP-GTE on improving the cognitive function in ovariectomized (OVX) rats. The LH paradigm has been proven to be a very useful technique to assess the cognitive dysfunction for understanding human depression^34^. To elucidate the mechanisms underlying the effects of HTP-GTE on PMD-induced cognitive dysfunction, we investigated the effect of HTP-GTE on the synaptic impairment at Schaffer collateral (SC)-CA1 synapses of the hippocampus from helpless OVX rats using electrophysiological and immuno-histochemical spine count techniques. We also examined whether GCG component itself can affect cognitive dysfunctions in helpless OVX rats by measuring spatial learning and memory using the Morris water maze test^35^. In this study, we demonstrated that HTP-GTE can prevent PMD-related cognitive impairments more effectively than conventional green tea (GTE). Furthermore, HTP-GTE was found to be functionally effective by restoring long-term synaptic plasticity. It induces the activation of silent synapses, which was mediated by the brain-derived neurotrophic factor (BDNF)/ tyrosine receptor kinase B (TrkB) signaling pathway. GCG was identified as a major bioactive constituent responsible for the main effect of HTP-GTE. Our findings suggested that GCG-enriched green tea derivatives are potential therapeutic candidates to prevent the development of cognitive deficits induced by PMD.

## Results

### HTP-GTE is safer and less neurotoxic than GTE

We developed HTP-GTE for the present study through a process involving epimerization that involved heating and pH adjustment^24^. The detailed process of manufacturing this modified green tea extract is described in the method section. To determine the bioavailability of HTP-GTE as a potential therapeutic for depression, we performed a toxicological investigation of HTP-GTE compared to the conventional green tea extract (GTE) under in vivo conditions. Either HTP-GTE or GTE was administered orally every day at a dose of 5 g/kg to a group of five male and five female rats that were fasted overnight. Mortality, clinical signs, body weights, and gross necropsy findings were evaluated. The results showed that there was no unscheduled death in HTP-GTE-treated rats. Three female rats and one of five male rats fed with GTE died during the study (Fig. S1a,b). Regarding the clinical signs and body weights, no treatment-related changes were observed in HTP-GTE-fed rats (Fig. S1c). At the end of the observation period all animals were euthanized by CO_2_ gas overdose. Any gross abnormalities in organs including kidney, liver, and spleen were observed with details of the location, color, shape, and size. No remarkable abnormalities were found at necropsy on the 15th day in HTP-GTE-fed rats (data not shown).

We also investigated the toxicity assay under in vitro conditions. By using 3-(4,5-dimethylthiazol-2-yl)-2,5-diphenyltetrazolium bromide (MTT) assay, we evaluated the neuronal cell viability in SH-SY5Y cells (human neuroblastoma cell line) and cultured rat hippocampal neurons following HTP-GTE or GTE treatment at various doses (3—100 μg/mL) for 2 h. In SH-SY5Y cells treated with GTE, cell viability decreased in a dose-dependent manner, but no significant difference in cell viability was observed between control and HTP-GTE-treated cells, except at high doses (100 μg/mL) (Fig. S1d). Similarly, GTE treatment significantly reduced hippocampal neuronal viability in a dose-dependent manner relative to control cells, but HTP-GTE administration had little effect on the neuronal viability, except at high doses (100 μg/mL) (Fig. S1e). These results strongly imply that HTP-GTE is a safer and less toxic green tea-based therapeutic than GTE.

### HTP-GTE administration improves resilience in OVX rats

To compare the effect of either HTP-GTE or GTE on the development of depression during menopause, we orally administered the green tea extracts in rats for 30 days before the LH induction protocol (Fig. S2c). Vehicle-fed OVX rats were firstly exposed to 50 randomly applied inescapable/unpredictable shocks for 3 days so that we could confirm the effectiveness of our shock protocol for LH induction in post-menopausal models. With these conditions, the same shock parameters were used for the rats fed with either HTP-GTE or GTE. On the 4th day of the LH protocol, the rats were tested for escape behavior to evaluate the induction of helplessness. Rats showing more than 20 escape failures during the course of 30 trials were referred to be "helpless"^36,37^. As shown in Fig. S2a–c, the shock protocol effectively induced LH with an incidence rate of 84.2% in vehicle-fed OVX rats (Fig. S2f–g). The administration of HTP-GTE almost completely suppressed the acquisition of LH in a dose-dependent manner in OVX rats (Fig. S2d–f). GTE (200 mg/kg) potently augmented resilience against the induction of LH in OVX rats to a similar degree of resilience seen with the same dose of HTP-GTE. However, GTE at a high dose (400 mg/kg) was not as effective as HTP-GTE in preventing the induction of LH in OVX rats (Fig. S2g–h), implying the superiority of HTP-GTE over GTE in attenuating the symptoms of PMD. It was imperative to minimize the contribution of HTP-GTE-independent resilience in the experiments involving HTP-GTE-treated OVX groups. Thus, we maximized the number of resilient rat populations by orally administering HTP-GTE at doses of 200 and 400 mg/kg, which almost completely prevented the induction of LH induction in OVX rats (Fig. S2h). These resilient rats were further categorized as HTP-GTE-fed OVX (OVX + HTP-GTE) group. To investigate the comparative effects of GTE and HTP-GTE on the synaptic alterations by LH in OVX rats, the same dose of GTE was administered, and the resilient rats selected were further categorized as GTE-fed OVX (OVX + GTE) group. For comparison, we used only “helpless” rats as vehicle-treated OVX (OVX) group. Sham-operated control (Sham resilient) is a group female rats which were exposed to foot shock chamber under the same LH induction protocol.

### Locomotor activities in OVX rats

To validate if there is any change in the motor function by surgical ovariectomy, rotarod test was performed. As shown in Fig. S3a, we confirmed that there was no change in the mobility of rats before and after ovariectomy. A complete ovariectomized state was also confirmed by enzyme-linked immunosorbent assay (ELISA) of 17β-estradiol (E2 estrogen) levels in serum. We measured the amount of E2 before and after ovariectomy and, as shown in Fig. S3b, OVX state is confirmed by lowered E2 levels in the serum after the surgery.

### HTP-GTE rescues the synaptic impairments induced by LH-induced stress via recovering LTP at Schaffer collateral-CA1 synapses in OVX rats

**Figure 1:**
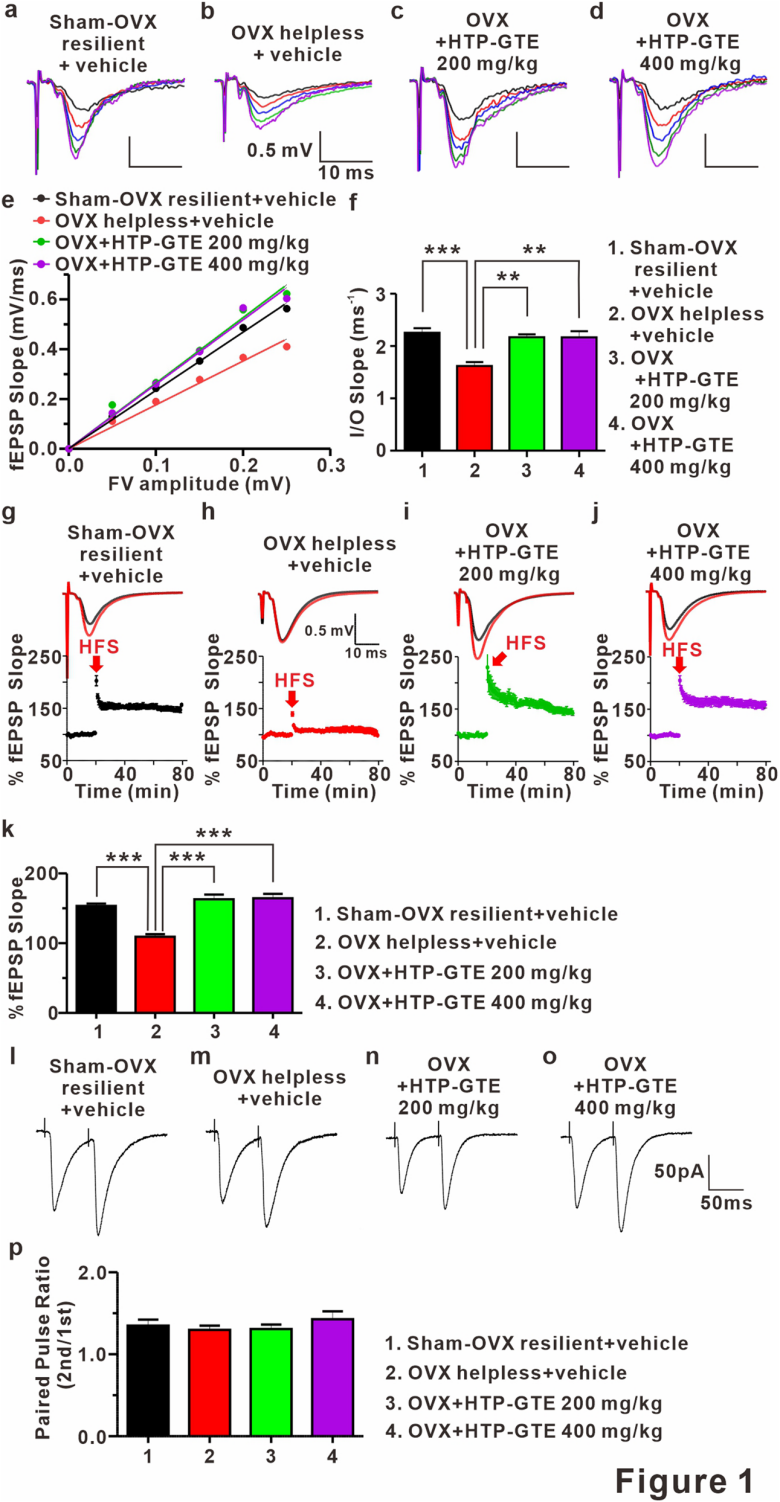
HTP-GTE rescues LH-induced synaptic impairments by restoring LTP at Schaffer collateral-CA1 synapses in OVX rats. (**a–d**) Representative traces of fEPSPs from hippocampal slices from representative experiments at four increasing stimulus intensities. (**e**) The scatter plot of the Input and Output (I/O) relationship corresponding to the recorded fEPSPs in A. (**f**) The average of slope I/O relationship for sham-OVX resilient + vehicle, OVX helpless + vehicle, OVX + HTP-GTE 200 mg/kg, and OVX + HTP-GTE 400 mg/kg group (Sham-OVX resilient + vehicle: 2.27 $\pm$ 0.07, n = 6 slices/3 rats; OVX helpless + vehicle: 1.62 $\pm$ 0.07, n = 6 slices/3 rats; OVX + HTP-GTE 200 mg/kg: 2.17 $\pm$ 0.06, n = 5 slices/3 rats; OVX + HTP-GTE 400 mg/kg: 2.22 $\pm$ 0.11, n = 5 slices/3 rats). (**g–j**) Top: representative traces showing field EPSPs before (average of 20 traces, black line) and after (average of 180 traces, red line) high-frequency stimulus. Bottom: average time courses for field EPSP amplitude during LTP induction in all groups. Data are shown as mean $\pm$ SEM. (**k**) Quantified graph was shown (Sham-OVX resilient + vehicle: 153.3 $\pm$ 3.39, n = 5 slices/3 rats; OVX helpless + vehicle: 109.2 $\pm$ 3.65, n = 6 slices/3 rats; OVX + HTP-GTE 200 mg/kg: 166.6 $\pm$ 6.66, n = 6 slices/3 rats; OVX + HTP-GTE 400 mg/kg: 143.3 $\pm$ 10.59, n = 6 slices/3 rats). (**l–o**) Representative traces of paired pulse-stimulation evoked EPSCs (50 Hz; average of 10 trials) for sham-OVX resilient + vehicle, OVX helpless + vehicle, OVX + HTP-GTE 200 mg/kg, OVX + GTE 200 mg/kg, OVX + HTP-GTE 400 mg/kg, and OVX + GTE 400 mg/kg. (**p**) Quantified graph was shown (Sham-OVX resilient + vehicle: 1.35 $\pm$ 0.07, n = 6 cells/3 rats; OVX helpless + vehicle: 1.29 $\pm$ 0.05, n = 6 cells/3 rats; OVX + HTP-GTE 200 mg/kg: 1.31 $\pm$ 0.06, n = 7 cells/3 rats; OVX + HTP-GTE 400 mg/kg: 1.43 $\pm$ 0.10, n = 8 cells/4 rats). Data are represented as mean $\pm$ SEM (One-way ANOVA/Tukey’s post hoc test, *p < 0.05, **p < 0.01, ***p < 0.001).

In an attempt to investigate the effect of HTP-GTE feeding on the synaptic alterations in the hippocampi of OVX rats induced by LH procedure, we compared the synaptic strengths and LTP induction at Schaffer collateral (SC)-CA1 circuits in the acute hippocampal brain slices by recording field excitatory postsynaptic potential (fEPSP). First, to rule out whether OVX-induced hormonal alteration may be directly involved in the effect of HTP-GTE on hippocampal synaptic function in helpless OVX rats, we prepared no shock control groups (Sham no shock, Sham no shock + HTP-GTE, OVX no shock, OVX no shock + HTP-GTE). The synaptic strengths and LTP induction were measured and compared between the groups. As shown in Fig. S4a–f, we confirmed that the synaptic strength at the SC-CA1 circuit was not attenuated in all the test groups with no shock. Similarly, LTP induction at SC-CA1 circuit was not suppressed in the OVX no shock group when compared with sham no shock group (Fig. S4g–k). These results strongly suggested that cognitive dysfunction due to PMD is not due to the hormone loss. Furthermore, we confirmed that the synaptic strength at the SC-CA1 circuit was significantly attenuated in helpless OVX rats compared to sham resilient controls (Figs. 1a,b,e,f, and S5a–d), which was consistent with a previous report^34^. This impaired synaptic strength was dramatically restored in HTP-GTE-fed OVX rats to the level exhibited by the sham resilient controls (Fig. 1c–f). Similarly, LTP induction at SC-CA1 circuit was significantly suppressed in the helpless OVX rats when compared with sham resilient controls (Figs. 1g,h,k and S5e–g), and this impairment was almost completely ameliorated in HTP-GTE-treated OVX rats (Fig. 1i–k). For comparison, we tested the LTP induction at SC-CA1 synapses in GTE-fed OVX rats. As shown in Fig. S6a–h, hippocampal LTP induction was also restored in GTE-fed OVX rats, but less potently than in the HTP-GTE-fed OVX group with the equivalent dose. To rule out the involvement of presynaptic alterations in the probability of transmitter release (Pr) in the HTP-GTE-induced synaptic improvements, the paired pulse ratio (PPR) was measured in the helpless OVX rats at SC-CA1 pyramidal cell synapses in all groups. As shown in Figs. 1i–p, and S5h–j there was no significant difference in PPR in all groups tested, indicating that the probability of presynaptic release was not altered by HTP-GTE feeding. These results suggest that Post-menopausal state is a "vulnerable factor” which makes the females be more susceptible to the stressors which may cause depression more easily as well as the associated cognitive impairments. In addition, HTP-GTE may ameliorate the hippocampal synaptic dysfunction due to the shock-triggered depression by the restoration of post-synaptic LTP in OVX rats.

### HTP-GTE augments the number of functional synapses without affecting synaptic strength at the hippocampal circuit in OVX rats

**Figure 2:**
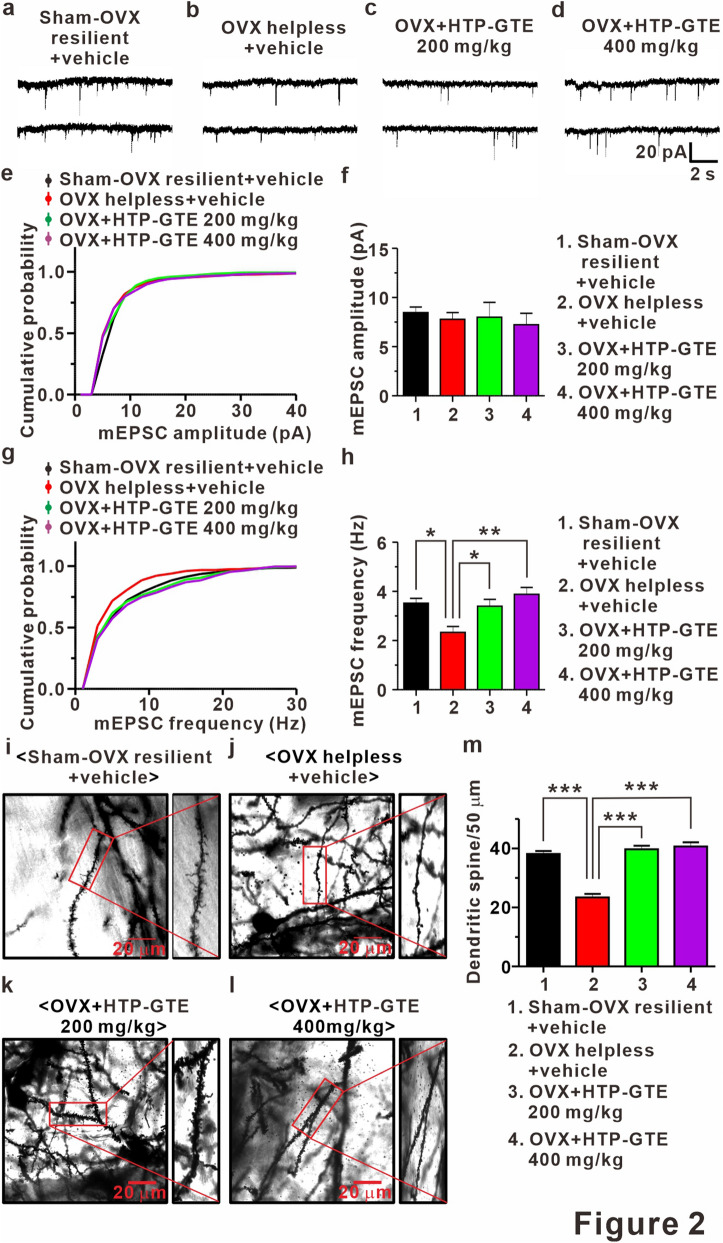
HTP-GTE augments the number of functional synapses without affecting synaptic strength at hippocampal circuit in OVX rats. (**a–d**) Sample traces showed miniature EPSCs for both groups. (**e**) Cumulative probability plots of mEPSC amplitude. mEPSC amplitude nearby sham resilient (Sham-OVX resilient + vehicle vs OVX helpless + vehicle: p = 0.9829; Sham-OVX resilient + vehicle vs OVX + HTP-GTE 200 mg/kg: p = 0.3581; Sham-OVX resilient + vehicle vs HTP-GTE 400 mg/kg: p = 0.9829, Kolmogorov–Smirnov two-sample test). (**f**) The mean amplitude of mEPSC for sham-OVX resilient + vehicle, OVX helpless + vehicle, OVX + HTP-GTE 200 mg/kg, and OVX + HTP-GTE 400 mg/kg group (Sham-OVX resilient + vehicle: 8.47 $\pm$ 0.57, n = 6 slices/3 rats; OVX helpless + vehicle: 7.78 $\pm$ 0.69, n = 7 slices/3 rats; OVX + HTP-GTE 200 mg/kg: 7.99 $\pm$ 1.52, n = 7 slices/3 rats; OVX + HTP-GTE 400 mg/kg: 7.24 $\pm$ 1.16, n = 6 slices/3 rats). (**g**) Cumulative probability plots of mEPSC frequency. mEPSC frequency nearby sham resilient (Sham-OVX resilient + vehicle vs OVX helpless + vehicle: p < 0.01; Sham-OVX resilient + vehicle vs OVX + HTP-GTE 200 mg/kg: p = 0.1863; Sham-OVX resilient + vehicle vs HTP-GTE 400 mg/kg: p = 0.557, Kolmogorov–Smirnov two-sample test). (**h**) The mean frequency of mEPSC for all groups (Sham-OVX resilient + vehicle: 3.52 $\pm$ 0.19, n = 6 slices/3 rats; OVX helpless + vehicle: 2.33 $\pm$ 0.24, n = 7 slices/3 rats; OVX + HTP-GTE 200 mg/kg: 3.39 $\pm$ 0.28, n = 7 slices/3 rats; OVX + HTP-GTE 400 mg/kg: 3.88 $\pm$ 0.28, n = 6 slices/3 rats). (**i–l**) Representative golgi-stained dendritic segments of CA1 pyramidal neuron from animals on sham-OVX resilient + vehicle, OVX helpless + vehicle, OVX + HTP-GTE 200 mg/kg, and OVX + HTP-GTE 400 mg/kg groups. (**m**) Quantitative analysis of spine density for all groups (Sham-OVX resilient + vehicle: 38.00 $\pm$ 1.47; OVX helpless + vehicle: 23.63 $\pm$ 1.89; OVX + HTP-GTE 200 mg/kg: 39.14 $\pm$ 2.25; OVX + HTP-GTE 400 mg/kg: 40.29 $\pm$ 2.99, 3–4 brains/group). Data are represented as mean $\pm$ SEM (One-way ANOVA/Tukey’s post hoc test *** p < 0.001).

New spines and associated synapses are formed following LTP induction in the hippocampus^38,39^, and LTP-induced synaptogenesis occurs only in newly-emerging spines that are in contact with axon terminals activated by LTP stimuli^39^. Therefore, it is possible that HTP-GTE may augment the synaptic strength, which is suppressed in helpless OVX rats, by improving the functional connectivity resulting from the restoration of LTP-induced synaptogenesis. To test this, we measured AMPA receptor-mediated miniature excitatory postsynaptic currents (mEPSCs) at CA1 pyramidal neurons in the acute hippocampal brain slices from each group. As shown in Figs. 2a,b,g,h and S6a,b,e,f the mean frequency of mEPSCs was significantly reduced in helpless OVX rats when compared with sham resilient controls. This attenuation of mEPSC frequency was significantly ameliorated in HTP-GTE-fed OVX rats (Fig. 2c,d,g,h). However, the mean amplitudes of mEPSCs were not altered in any of the experimental groups (Figs. 2a–f and S6a–d). Furthermore, CA1 dendritic spine density was significantly decreased in helpless OVX rats when compared with sham resilient controls, consistent with the previous report (Figs. 2i,j,m and S6g–i)^34^. Reduced spine density was almost completely recovered to sham resilient control levels in HTP-GTE-fed OVX rats (Fig. 2i–m). These results strongly suggest that HTP-GTE ameliorates the synaptic impairments by increasing a new functional connectivity, but not by potentiating the synaptic strength in already existing functional synapses.

### Silent synapses re-emerge following HTP-GTE administration in OVX rats

**Figure 3:**
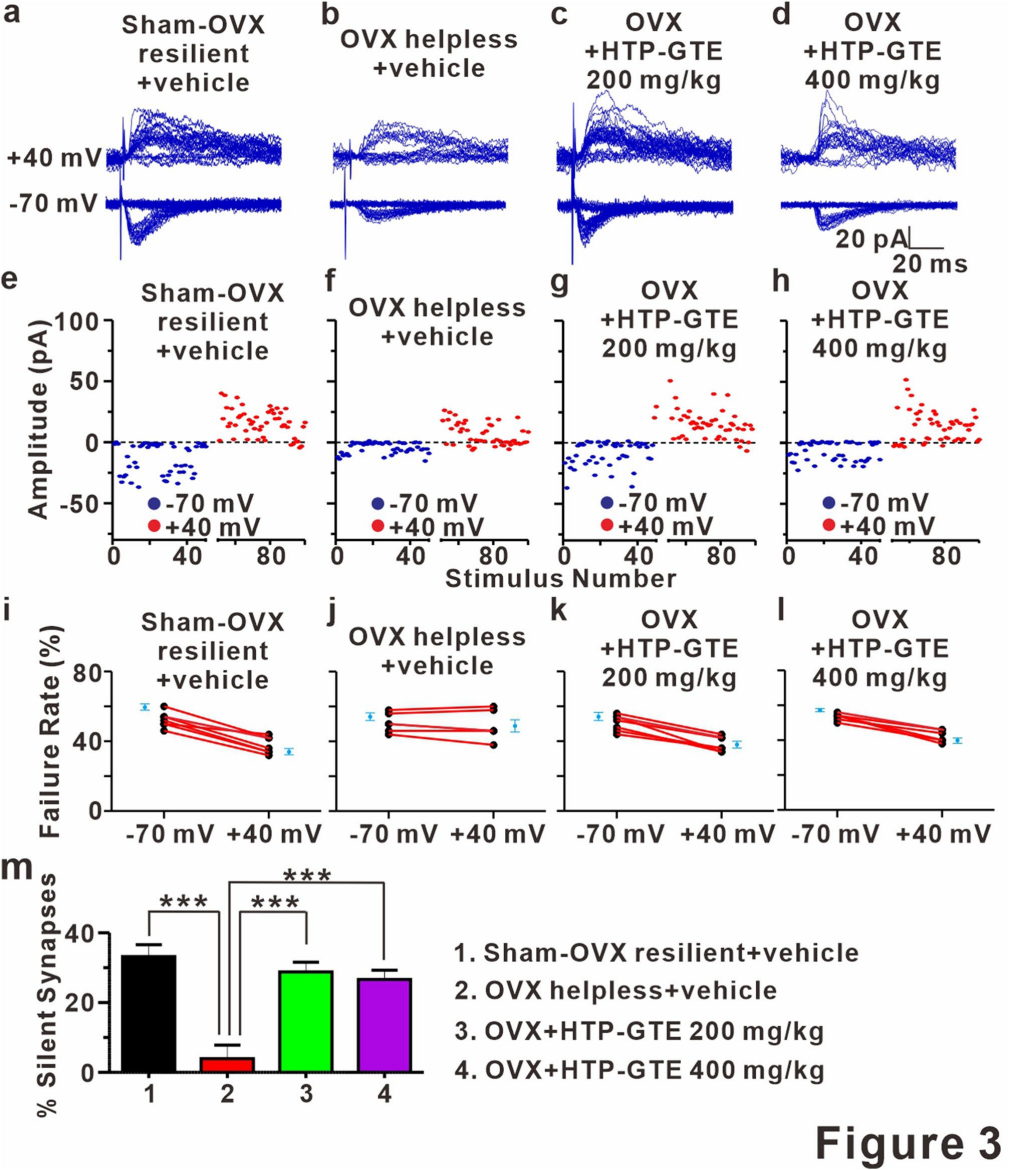
Silent synapses are re-emerged following HTP-GTE administration in OVX rats. (**a-d**) Representative traces for EPSCs evoked by minimal stimulation for 50 trials at holding potentials of -70 mV or + 40 mV in the slices from sham-OVX resilient + vehicle, OVX helpless + vehicle, OVX + HTP-GTE 200 mg/kg, and OVX + HTP-GTE 400 mg/kg groups. (**e–h**) Time course of EPSC amplitudes in representative cells shown in collected -70 mV (blue symbols) and + 40 mV (red symbols). (**i–l**) Failure rates for EPSCs at − 70 mV or + 40 mV in slices for all groups. (**m**) Percentage of silent synapse proportions for all groups (Sham-OVX resilient + vehicle: 33.19 $\pm$ 3.38, n = 7; OVX helpless + vehicle: 3.925 $\pm$ 3.89, n = 6; OVX + HTP-GTE 200 mg/kg: 28.78 $\pm$ 2.82, n = 7; OVX + HTP-GTE 400 mg/kg: 26.63 $\pm$ 2.67, n = 6).

A silent synapse is defined as one possessing post-synaptic NMDA receptors, but no AMPA receptors, which makes it unable to mediate synaptic transmission under physiological conditions^40–42^. Silent synapses occur in early developmental stage (critical period) and advanced aging, serving as a reservoir for experience-dependent synaptic plasticity, including LTP, in hippocampus^43^. LTP converts silent synapses into functional ones by inserting AMPA receptor into the postsynaptic membrane^40–42^. In addition, LTP-induced functionalization of silent synapses usually results in an increase in the number of functional synapses (i.e. increase in mEPSC frequency) without altering presynaptic release probability^44,45^, which is consistent with the effect of HTP-GTE on the quantal contents in SC-CA1 pyramidal cell synapses shown in Figs. 2 and S7. Therefore, it is possible that the reemergence of silent synapses may contribute to HTP-GTE-induced restoration of LTP induction, which is suppressed in helpless OVX rats. To test this hypothesis, the proportion of silent synapses was measured using a minimal stimulation protocol by comparing failure rates at holding potentials of − 70 mV and + 40 mV^42^. As shown in Figs. 3a, b,e,f,i,j and S8 the silent synapses were present at SC-CA1 synapses in the brain slices from sham resilient control but were significantly reduced in those from helpless OVX rats. Reduction in silent synapse formations in the helpless OVX rats was significantly reversed by HTP-GTE administration (Fig. 3c,d,g,h,k,l,m). We confirmed this result by measuring silent synapses through a minimal stimulation intensity at which no AMPA EPSCs were detected (at − 70 mV) and a subsequent depolarization of the target neuron to + 40 mV, so that we could detect NMDA-only EPSCs^40,46,47^. NMDA-only EPSCs were detected in all SC-CA1 synapses recorded in sham resilient controls, but not in helpless OVX rats (Fig. S9a,b,e,f,i). The suppression of NMDA-only EPSCs was significantly abrogated by HTP-GTE application in the OVX models (Fig. S9c,d,g,h,i), implying a possible role of the activated silent synapses by HTP-GTE-induced recovery of the long-term plasticity impairments in OVX rats.

### Regulation of BDNF-TrkB pathway underlies the HTP-GTE-dependent amelioration of hippocampal synaptic impairments in helpless OVX rats

**Figure 4:**
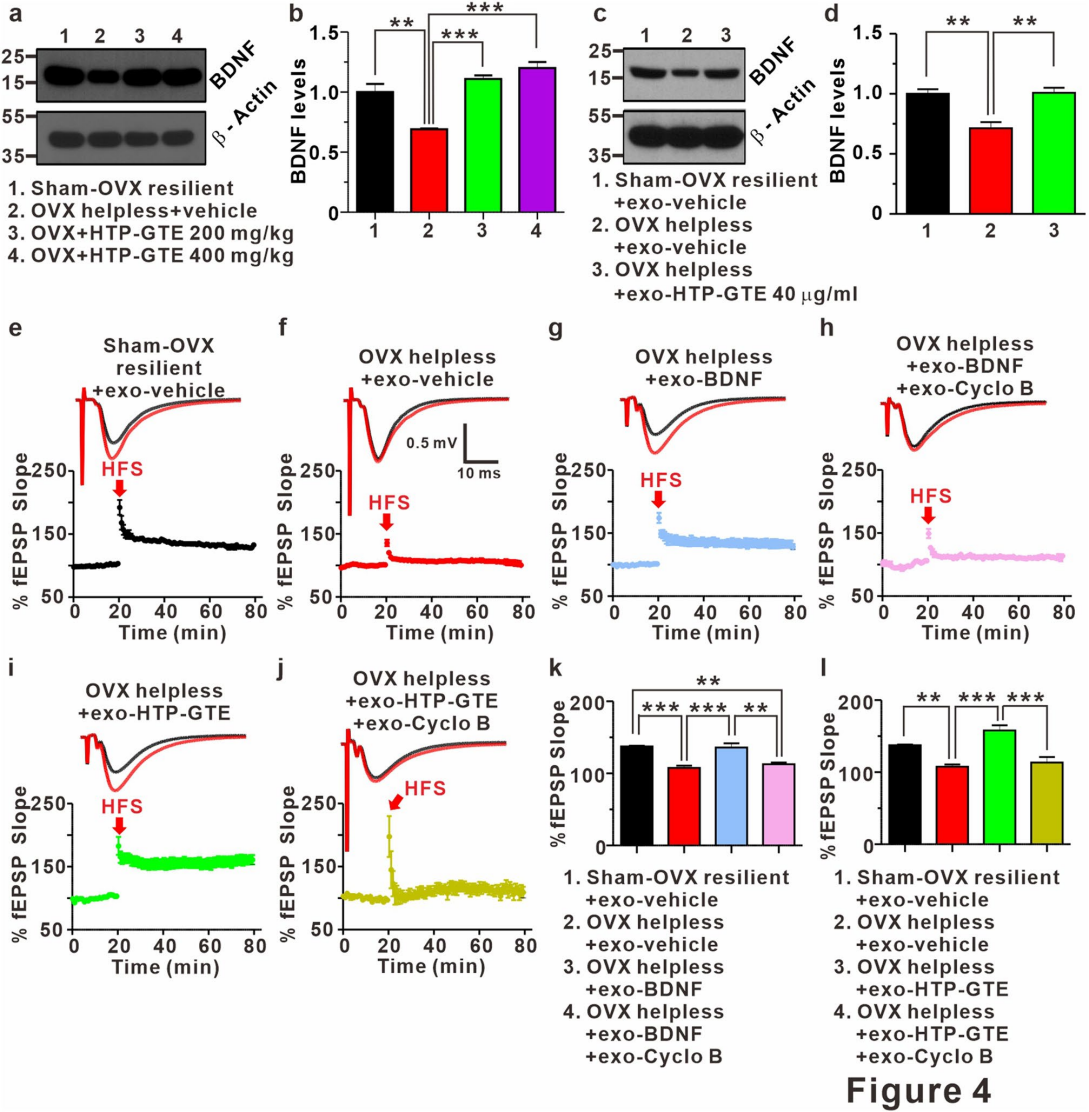
Regulation of BDNF-TrkB pathway underlies HTP-GTE-dependent amelioration of hippocampal synaptic impairments in helpless OVX rats. (**a, b**) Western blot of BDNF level in hippocampus on sham-OVX resilient + vehicle, OVX helpless + vehicle, OVX + HTP-GTE 200 mg/kg, and OVX + HTP-GTE 400 mg/kg groups in oral administration of HTP-GTE or vehicle solution daily for 4 weeks (Sham-OVX resilient + vehicle: 1.00 $\pm$ 0.07; OVX helpless + vehicle: 0.69 $\pm$ 0.01; OVX + HTP-GTE 200 mg/kg: 1.11 $\pm$ 0.03; OVX + HTP-GTE 400 mg/kg: 1.20 $\pm$ 0.05, n = 3 brains/group). Full-length blot is presented in Supplementary Fig. S13. (**c,d**) Western blot of BDNF level in hippocampus of exogenous application of HTP-GTE for 2 h (Sham-OVX resilient + exo-vehicle: 1.0 $\pm$ 0.04; OVX helpless + exo-vehicle: 0.71 $\pm$ 0.05; OVX helpless + exo-HTP-GTE 40 μg/ml: 1.01 $\pm$ 0.04, n = 3 brains/group). Full-length blot is presented in Supplementary Fig. S13. (**e–j**) Top: representative traces showing EPSPs before (average of 20 traces, black line) and after (average of 180 traces, red line) high-frequency stimulus. Bottom: average time courses for EPSP amplitudes during LTP induction in all groups. (**k,l**) Quantified graph was shown (Sham-OVX resilient + exo-vehicle: 137.4 $\pm$ 1.02, n = 5 slices/3 rats; OVX helpless + exo-vehicle: 107.6 $\pm$ 3.18, n = 8 slices/3 rats; OVX helpless + exo-BDNF (8 nM): 136.0 $\pm$ 5.74, n = 6 slices/3 rats; OVX helpless + exo-BDNF (8 nM) + exo-cyclo B: 112.8 $\pm$ 2.27, n = 6 slices/3 rats; OVX helpless + exo-HTP-GTE 40 μg/ml: 157.9 $\pm$ 7.21, n = 7; OVX helpless + exo-HTP-GTE 40 μg/ml + cyclo B: 113.4 $\pm$ 7.64, n = 6). Data are represented as mean $\pm$ SEM (One-way ANOVA Tukey’s post hoc test, **p < 0.01, ***p < 0.001).

BDNF has been known as an important regulator of long-term synaptic plasticity processes in the hippocampus underlying learning and memory during adulthood^48–50^. Down-regulation of BDNF levels in the hippocampus plays an important role in depression-related symptoms, including cognitive impairment^51–53^. Therefore, it is possible that the amelioration of hippocampal synaptic impairment induced by HTP-GTE may be mediated by increased BDNF levels, resulting in reversing LTP impairments in helpless OVX rats. To test this hypothesis, we compared the hippocampal BDNF expression in the helpless OVX and HTP-GTE-fed OVX rats using an immunoblot. In addition, we tested the effect of exogenous HTP-GTE treatment on BDNF expressions in acute hippocampal slices from the helpless OVX rats. As shown in Fig. 4a,b, and Fig. S10a–d the hippocampal BDNF expression was significantly suppressed in helpless OVX rats when compared with sham resilient controls. Suppressed BDNF expression was recovered to sham resilient control levels in HTP-GTE-treated OVX rats. Moreover, surprisingly, the exogenous treatment of HTP-GTE (40 μg/mL) for 2 h significantly augmented BDNF expression in the acute hippocampal slices from the helpless OVX rats when compared with sham resilient controls (Fig. 4c,d). We further tested the effect of exogenous BDNF and HTP-GTE on LTP induction at SC-CA1 synapses in acute hippocampal slices from the helpless OVX rats. As shown in Fig. 4e–h,k, exogenous BDNF application effectively ameliorated the impairment of LTP induction at SC-CA1 synapses in helpless OVX rats. The ameliorating effect of exogenous BDNF on hippocampal LTP was blocked by co-treatment with 1 μM cyclotraxin B (cyclo B), a potent inhibitor of TrkB^54^. Similar to BDNF effect, the exogenous application of HTP-GTE (40 μg/mL) rescued the impairments of LTP at SC-CA1 synapses in the acute hippocampal slices from the helpless OVX rats, however, this recovery by HTP-GTE was nearly completely prevented by 1 μM cyclo B (Fig. 4i,j,l). These results suggest that HTP-GTE may improve depression-related cognitive dysfunction by restoring LTP via the activation of the BDNF-TrkB signaling pathway.

### BDNF-TRkB-mediated signaling contributes to the reemergence of silent synapses in HTP-GTE-treated OVX rats

**Figure 5:**
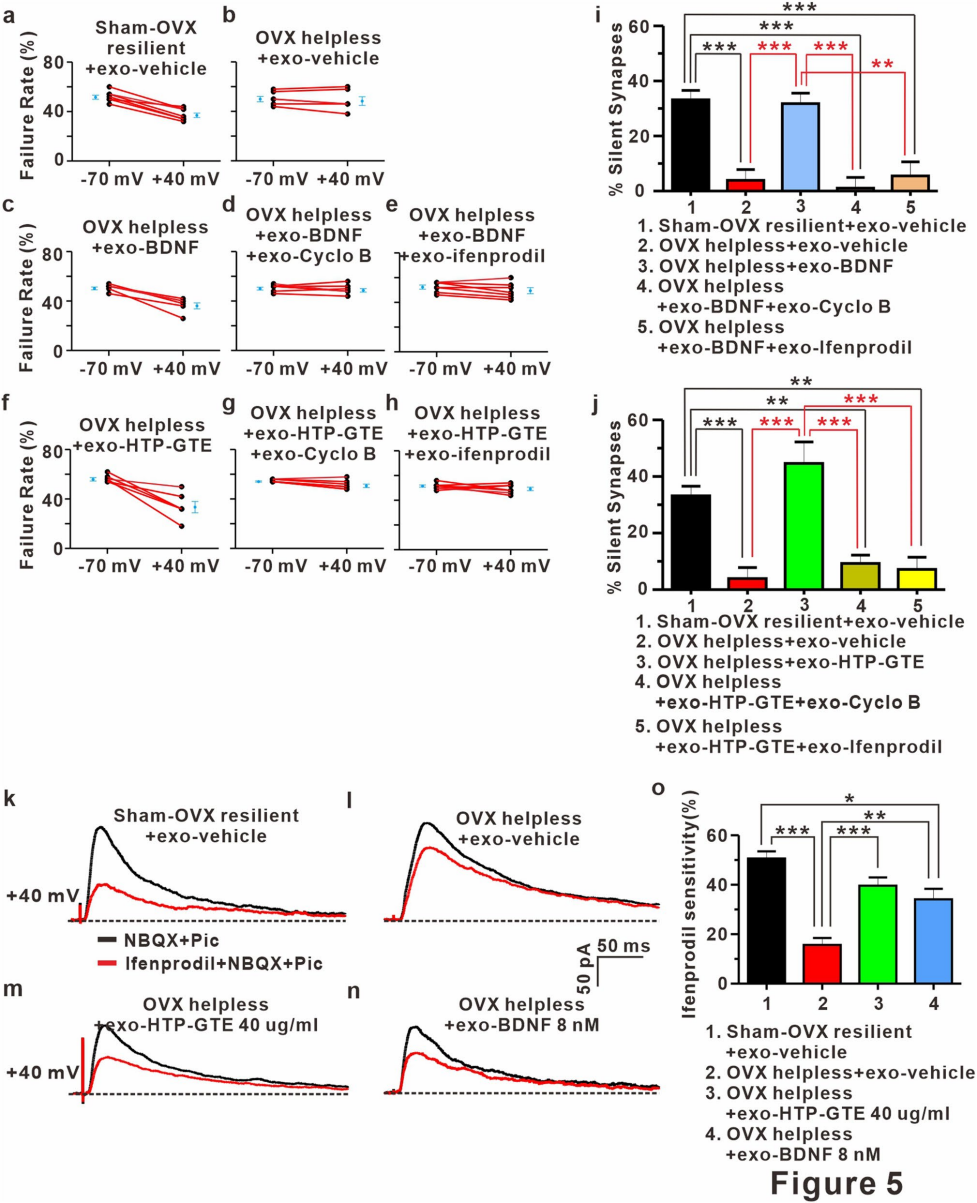
BDNF-mediated augmentation of GluN2B expressions contributes to the activation of silent synapses in HTP-GTE-treated OVX rats. (**a–h**) Failure rates for EPSCs at -70 mV or + 40 mV in slices from sham-OVX resilient + exo-vehicle, OVX helpless + exo-vehicle, OVX helpless + exo-BDNF, OVX helpless + exo-BDNF + exo-cyclo B, OVX helpless + exo-BDNF + ifenprodil, OVX helpless + exo-HTP-GTE, OVX helpless + exo-HTP-GTE + exo-cyclo B, and OVX helpless + exo-HTP-GTE + exo-ifenprodil. (**i,j**) Summary of the percentage of silent synapse for all groups (Sham-OVX resilient + exo-vehicle: 33.19 $\pm$ 3.38, n = 7; OVX helpless + exo-vehicle: 3.93 $\pm$ 3.89, n = 6; OVX helpless + exo-BDNF: 31.79 $\pm$ 3.80, n = 6; OVX helpless + exo-BDNF + exo-cyclo B: 1.08 $\pm$ 3.88, n = 7; OVX helpless + exo-BDNF + exo-ifenprodil: 5.52 $\pm$ 5.12, n = 7; OVX helpless + exo-HTP-GTE: 44.61 $\pm$ 7.64, n = 6; OVX helpless + exo-HTP-GTE + exo-cyclo B: 9.30 $\pm$ 2.93, n = 7; OVX helpless + exo-HTP-GTE + exo-ifenprodil: 7.18 $\pm$ 4.28, n = 7). (**k–n**) Representative traces for the effect of infeprodil on the NMDA EPSCs in hippocampal slices from sham-OVX resilient + exo-vehicle, OVX helpless + exo-vehicle, OVX helpless + exo-HTP-GTE, and OVX helpless + exo-BDNF groups. **o**. Percentage of ifenprodil-sensitivity current for all groups (Sham-OVX resilient + exo-vehicle: 50.62 $\pm$ 2.91, n = 6; OVX helpless + exo-vehicle: 15.68 $\pm$ 2.77, n = 5; OVX helpless + exo-HTP-GTE 40 μg/ml: 39.61 $\pm$ 3.35, n = 6; OVX helpless + exo-BDNF 8 nM: 34.11 $\pm$ 4.26, n = 5).

Functionalization of silent synapses is a principal mechanism for NMDAR dependent LTP, and BDNF/TrkB-mediated signaling is required for functionalization of silent synapses^55–57^. In addition, GluN2B-containing NMDAR plays a critical role in the formation and maintenance of silent synapses^58,59^. Therefore, it is possible that BDNF-TrkB signaling may be involved in the HTP-GTE-mediated reemergence of silent synapses in the hippocampus in the helpless OVX rats. This was checked by determining the effect of cyclo B (1 μM) and ifenprodil (5 μM: a potent GluN2B antagonist) on HTP-GTE-induced reemergence of silent synapses at SC-CA1 circuits using the same protocol as in Fig. 3 in the acute hippocampal slices taken from helpless OVX rats. Co-treatment with cyclo B or ifenprodil effectively prevented HTP-GTE-induced reemergence of silent synapses in helpless OVX rats (Fig. 5a,b,f–h,j). Similar to the results of HTP-GTE treatment, the exogenous BDNF significantly increased the number of silent synapses at SC-CA1 circuits as seen in the studies with the acute hippocampal slices from the helpless OVX rats (Fig. 5a–e,i). The effect of BDNF on the proportion of silent synapses was nearly completely inhibited by co-treatment with cyclo B or ifenprodil (Fig. 5d,e,i). In term to the effect of ifenprodil, it is also possible that it could be that new silent synapses preferentially have GluN2B and so adding ifenprodil mean it can no longer detect them even if they still exist in newly formed silent synapses. To rule out this possibility, we evaluated ifenprodil sensitivity with the percentage of GluN2B-containing NMDA receptor by calculating the changes in the responses before and after the administration of drug in order to functionally confirm the HTP-GTE- or BDNF-induced increase of GluN2B expression at SC-CA1 circuits in acute hippocampal slices from the helpless OVX rats. Furthermore, we investigated whether direct ex vivo application of HTP-GTE or BDNF could cause an increase in the functional numbers of dendritic spines by measuring the mEPSC frequency in the acute hippocampal slices from the helpless OVX rats. In the helpless OVX rat, NMDA EPSCs at SC-CA1 circuits confirmed a decrease in ifenprodil sensitivity when compared with sham-OVX resilient controls. This decrease of ifenprodil sensitivity was significantly restored to the levels of sham-OVX resilient controls after the exogenous HTP-GTE- or BDNF- treatment (Fig. 5k–o). In addition, the suppression of mEPSC frequencies by LH was recovered to the levels of sham-OVX resilient control in the acute hippocampal slices from helpless OVX rats treated with exogenous HTP-GTE (40 μg/mL) or BDNF (8 nM) (Fig. S11a–c,j–l,h–i,q–r). However, the mean amplitudes of mEPSCs were not altered by HTP-GTE or BDNF administration, and this result was demonstrated in the acute hippocampal slices from helpless OVX rats (Fig. S11a–c,j–l,f–g,o–p). Furthermore, the ameliorating effect of exogenous BDNF or HTP-GTE on the suppressed mEPSC frequencies was blocked by co-treatment with cyclo B or ifenprodil (Fig. S11d–i,m–o,r), which implied the involvement of GluN2B-containing NMDA receptors in HTP-GTE-induced increase of functional synaptic connection in helpless OVX rats.

### Expression levels of BDNF and GluN2B at hippocampus in HTP-GTE treated-OVX rats

**Figure 6:**
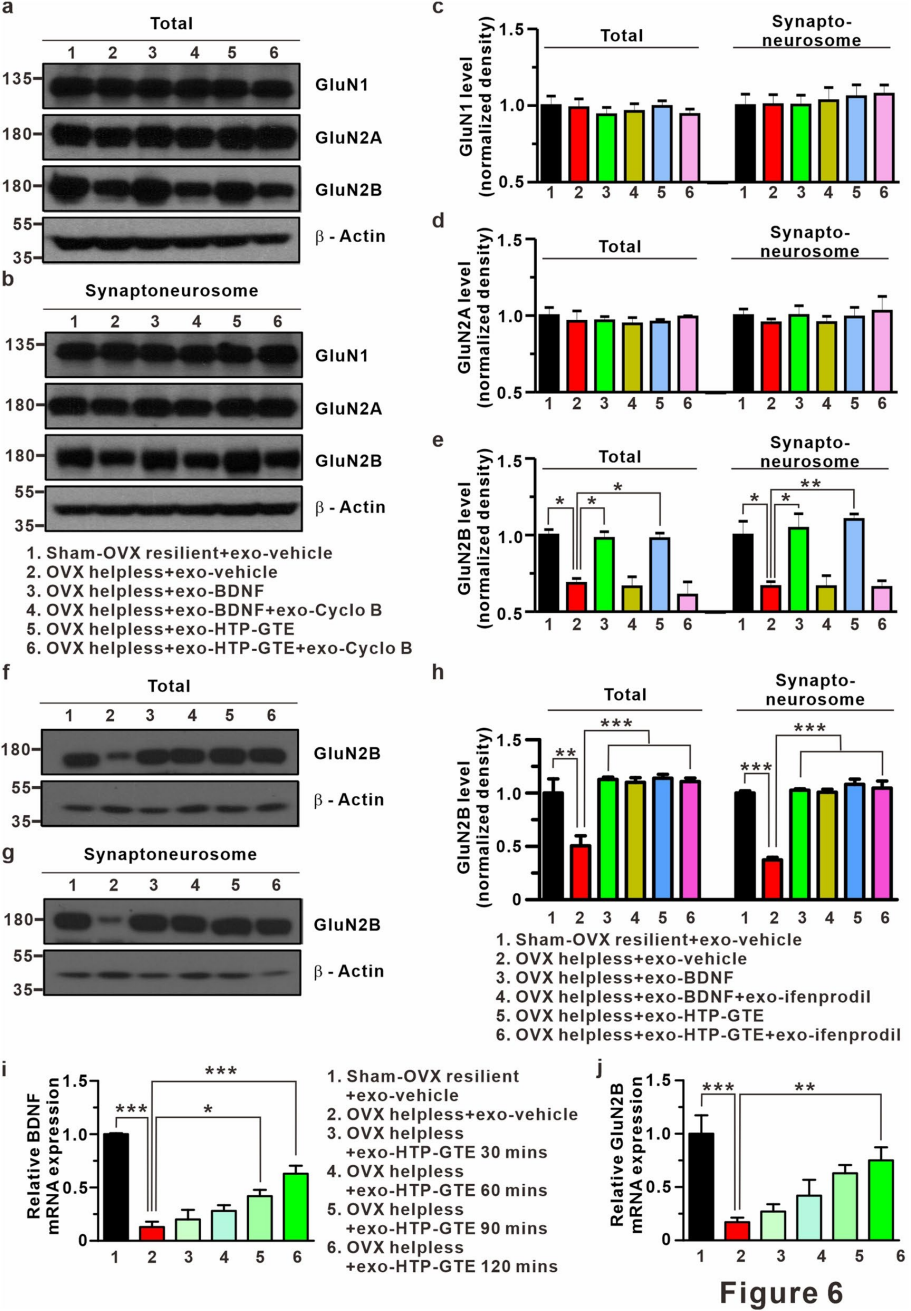
BDNF-mediated augmentation of GluN2B expression contributes to activation of silent synapses in HTP-GTE-treated OVX rats. (**a,b**) Western blot of GluN1, GluN2A, GluN2B levels in brain lysate for total and synaptoneurosomal fraction from hippocampus. Full-length blots are presented in Supplementary Fig. S13. (**c–e**) Quantification of GluN1, GluN2A, GluN2B levels (GluN2B, total: Sham-OVX resilient + exo-vehicle: 1.00 $\pm$ 0.04; OVX helpless + exo-vehicle: 0.69 $\pm$ 0.03; OVX helpless + exo-BDNF: 0.98 $\pm$ 0.04; OVX helpless + exo-BDNF + exo-cyclo B: 0.66 $\pm$ 0.07; OVX helpless + exo-HTP-GTE: 0.98 $\pm$ 0.04; OVX helpless + exo-HTP-GTE + exo-cyclo B: 0.61 $\pm$ 0.09; Synaptoneurosome: Sham-OVX resilient + exo-vehicle: 1.00 $\pm$ 0.09; OVX helpless + exo-vehicle: 0.66 $\pm$ 0.03; OVX helpless + exo-BDNF: 1.04 $\pm$ 0.09; OVX helpless + exo-BDNF + exo-cyclo B: 0.66 $\pm$ 0.07; OVX helpless + exo-HTP-GTE: 1.10 $\pm$ 0.04; OVX helpless + exo-HTP-GTE + cyclo B: 0.66 $\pm$ 0.04, n = 3 brains/group). (**f,g**) Western blot of GluN2B levels in brain lysate for total and synaptoneurosomal fraction from hippocampus. Full-length blots are presented in Supplementary Fig. S13. (**h**) Quantification of GluN2B levels (GluN2B, total: Sham-OVX resilient + exo-vehicle: 1.00 $\pm$ 0.13; OVX helpless + exo-vehicle: 0.51 $\pm$ 0.09; OVX helpless + exo-BDNF: 1.13 $\pm$ 0.02; OVX helpless + exo-BDNF + exo-ifenprodil: 1.11 $\pm$ 0.04; OVX helpless + exo-HTP-GTE: 1.14 $\pm$ 0.04; OVX helpless + exo-HTP-GTE + ifenprodil: 1.11 $\pm$ 0.03; Synaptoneurosome: Sham-OVX resilient + exo-vehicle: 1.00 $\pm$ 0.02; OVX: 0.37 $\pm$ 0.02; OVX helpless + exo-BDNF: 1.01 $\pm$ 0.01; OVX helpless + exo-BDNF + exo-ifenprodil: 1.01 $\pm$ 0.03; OVX helpless + exo-HTP-GTE: 1.08 $\pm$ 0.05; OVX helpless + exo-HTP-GTE + exo-ifenprodil: 1.05 $\pm$ 0.07, n = 3 brains/group). (**i**,**j**) Quantitative real-time PCR. Data represent the BDNF and GluN2B gene expressions normalized to GAPDH gene. Values are mean $\pm$ SEM of three independent experiments. Data are represented as mean $\pm$ SEM (One-way ANOVA Tukey’s post hoc test, *p < 0.05, **p < 0.01, ***p < 0.001).

With the electrophysiological evidences supporting the involvement of GluN2B in BDNF-TrkB signaling, we also found that the exogenous BDNF or HTP-GTE increased the expression of GluN2B proteins without altering GluN1 or GluN2A expressions in the hippocampal synaptosomes from the helpless OVX rats. HTP-GTE- or BDNF-mediated increase in GluN2B expression was significantly suppressed by cyclo B co-treatment (Fig. 6a–e). However, HTP-GTE- or BDNF-mediated increase GluN2B expression was not suppressed by ifenprodil co-treatment (Fig. 6f–h). We also assessed the role of HTP-GTE in the genomic levels of BDNF and GluN2B by real-time quantitative polymerase chain reaction (qPCR), which was conducted to quantify the time-dependent mRNA expression levels at every 30 min. The effect of exogenous HTP-GTE treatment on the acute brain slice was observed through the genomic expression levels of target genes, and the expression of BDNF was detected at 90 min after the drug administration while GluN2B was detected at 120 min (Fig. 6i,j). These results suggested that BDNF-TrkB signaling contributes to HTP-GTE-activated silent synapses by regulating GluN2B-containing NMDARs.

### GCG, not EGCG, is a major contributor to the HTP-GTE-induced improvement of synaptic and cognitive impairments in the helpless OVX rats

**Figure 7:**
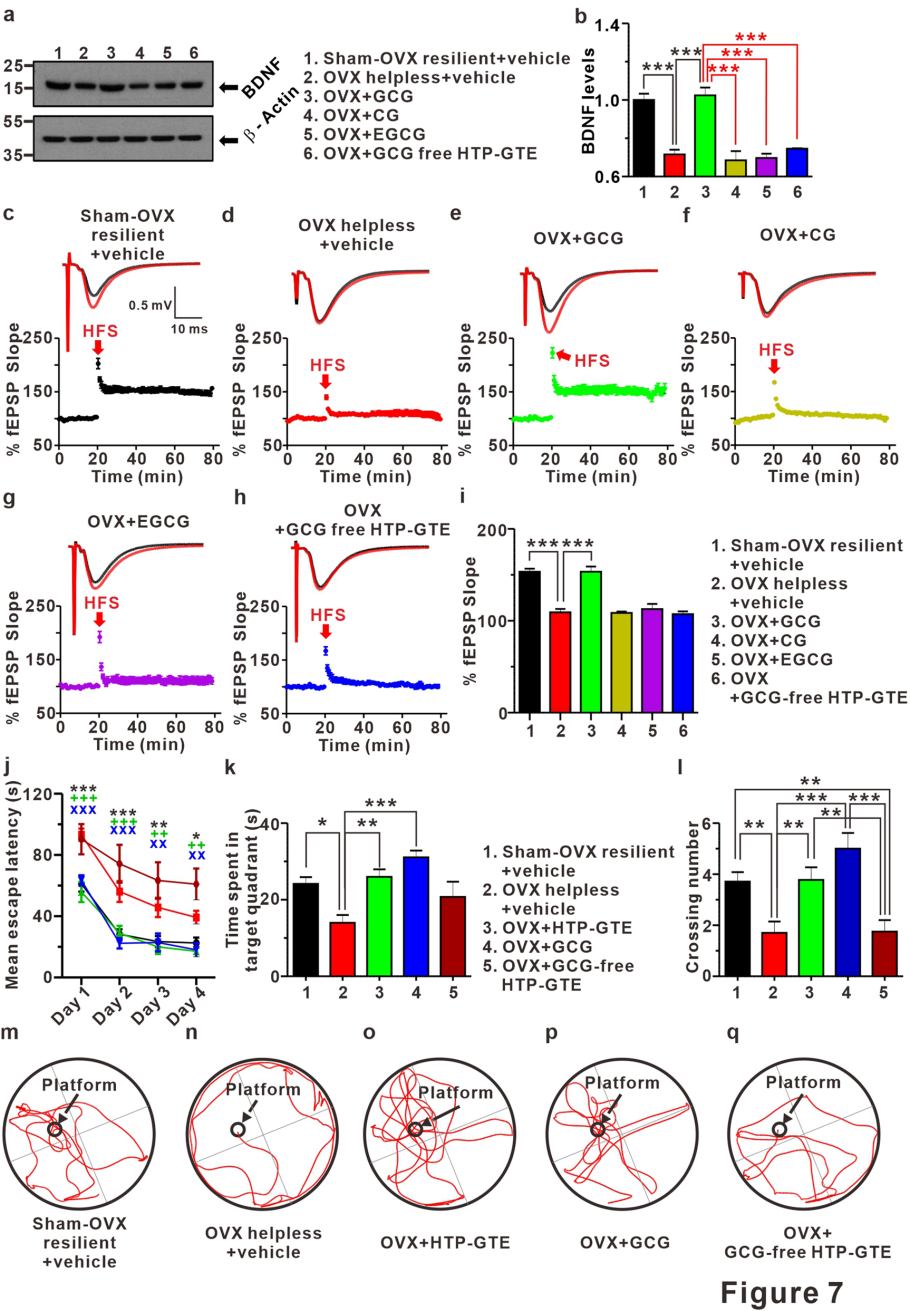
GCG, not EGCG, plays a role as a major contributor to HTP-GTE-induced improvement of synaptic and cognitive impairments in helpless OVX rats. (**a**,**b**) Western blot of BDNF level in the hippocampi of sham-OVX resilient + vehicle, OVX helpless + vehicle, OVX + GCG, OVX + CG, OVX + EGCG, and OVX + GCG-free HTP-GTE via oral administration of each green tea component daily for 4 weeks (Sham-OVX resilient + vehicle: 1.00 $\pm$ 0.03; OVX helpless + vehicle: 0.72 $\pm$ 0.02; OVX + GCG: 1.02 $\pm$ 0.04; OVX + CG: 0.69 $\pm$ 0.05; OVX + EGCG: 0.70 $\pm$ 0.02; OVX + GCG free HTP-GTE: 0.75 $\pm$ 0.02, n = 3 brains/group). Full-length blot is presented in Supplementary Fig. S13. (**c–h**) Top: representative traces showing EPSPs before (average of 20 traces, black line) and after (average of 180 traces, red line) high-frequency stimulus. Bottom: average time courses for EPSP amplitude during LTP induction on all groups. (**i**) Quantified graph was shown (Sham-OVX resilient + vehicle: 153.3 $\pm$ 3.39, n = 5 slices/3 rats; OVX helpless + vehicle: 109.2 $\pm$ 3.65, n = 6 slices/3 rats; OVX + GCG: 153.4 $\pm$ 5.72, n = 6 slices/3 rats; OVX + CG: 108.5 $\pm$ 1.51, n = 6 slices/3 rats; OVX + EGCG: 112.8 $\pm$ 5.58, n = 6 slices/3 rats; OVX + GCG free HTP-GTE: 107.3 $\pm$ 2.96, n = 6 slices/3 rats). (**j**) Morris water maze learning curves of 4 consecutive days. Data points represent mean value of escape latency of each day (Two-way repeated measured (RM) ANOVA Tukey’s post hoc test, *p < 0.05, **p < 0.01, ***p < 0.001). (**k**) Quantification of time spent in target quadrant (Sham-OVX resilient + vehicle: 24.16 $\pm$ 1.75, n = 14; OVX helpless + vehicle: 14.00 $\pm$ 2.01, n = 10; OVX + HTP-GTE: 25.99 $\pm$ 1.97, n = 9; OVX + GCG: 31.07 $\pm$ 1.78, n = 7; OVX + GCG free HTP-GTE: 20.74 $\pm$ 3.98, n = 8). (**l**) Quantification of crossing number (Sham-OVX resilient + vehicle: 3.71 $\pm$ 0.37, n = 14; OVX helpless + vehicle: 1.7 $\pm$ 0.45, n = 10; OVX + HTP-GTE: 3.78 $\pm$ 0.49, n = 9; OVX + GCG: 5.00 $\pm$ 0.62, n = 7; OVX + GCG free HTP-GTE: 1.75 $\pm$ 0.45, n = 8). Data are represented as mean $\pm$ SEM (One-way ANOVA Tukey’s post hoc test, *p < 0.05, **p < 0.01, ***p < 0.001). (**m–q**) Representative Morris water maze movement track from all groups.

We developed HTP-GTE for the present study through a process involving epimerization that involved heating and pH adjustment^24^. To compare the compositions of catechins in HTP-GTE and GTE in detail, we analyzed the constituents of both green tea derivatives by Ultra-performance liquid chromatography photometric diode array (UPLC-PDA). As shown in Table S1, EGCG and EGC were the major catechins present in both conventional green tea (GTE) and modified green tea (HTP-GTE). However, the levels of GCG and CG (i.e. epimers of EGCG and EGC, respectively) were much higher in HTP-GTE than in GTE. Firstly, each rat was individually fed with GCG, CG, EGCG, or HTP-GTE containing no GCG (i.e. GCG-free HTP-GTE), and exposed to the protocol shown in Fig. S2, so that we can independently assess the effect of each HTP-GTE catechin component on the LH models of OVX rats. Each animal group was subsequently tested for the escape behaviors to evaluate the induction of LH. As shown in Fig. S12a,b, the administration of GCG and EGCG, and not CG or GCG-free HTP-GTE, significantly increased the rodent resilience against LH in OVX rats. This is an important finding to primarily indicate that HTP-GTE-induced resilience against LH in OVX rats is due to either GCG or EGCG. Therefore, in the current study, we designed our experimental protocols to use the resilient rats only in the GCG- or EGCG-fed groups. Meanwhile, the helpless OVX rats were enrolled into two groups to study the CG-only HTP-GTE or GCG-free HTP-GTE-fed effects. To identify the major active components of the HTP-GTE effect, we investigated the effects of equivalent doses of GCG (5.58 mg/kg), CG (0.64 mg/kg), and EGCG (25 mg/kg) present in HTP-GTE on the hippocampal BDNF expressions and LTP induction in OVX rats, which were fed with the individual components for 30 days. Consistent with the results shown in Figs. 4a,b and 7a,b showed that the hippocampal BDNF expression was significantly suppressed in the helpless OVX rats when compared with sham-OVX resilient controls. Notably, only GCG-fed OVX rats exhibited a significant recovery of the suppressed hippocampal BDNF expressions while neither CG-fed nor EGCG-fed OVX rats showed any changes in BDNF expressions. In addition, GCG-free HTP-GTE-fed OVX rats showed no statistically significant alteration in the hippocampal BDNF expressions when compared with the helpless OVX rats (Fig. 7a,b). Administration of GCG nearly completely rescued impairment of LTP induction at SC-CA1 synapses in OVX rats, but CG, EGCG, and GCG-free HTP-GTE treatment showed little effect (Fig. 7c–i). Similarly, the exogenous administration of GCG restored LTP induction to sham-OVX resilient control levels, as observed in the acute hippocampal slices from the helpless OVX rats. These results suggested that both GCG and EGCG are effective in promoting and maintaining resilience against the induction of depression by LH protocol, but GCG on its own was by far more potent in recovering the depression-related synaptic impairments than EGCG. In an attempt to further evaluate the effects of HTP-GTE and its major active component, GCG, on memory and learning (which are usually malfunctioning during depression), all animal groups were trained to find the hidden platform in a water pool for 4 days, and the Morris water maze test was performed on the 5th day. As shown in Fig. 7j–l,m–q, the helpless OVX rats spent more time to find the hidden platform and performed fewer platform crossings than sham-OVX resilient controls. On the other hand, the oral administration of HTP-GTE or GCG in the OVX rats significantly restored their ability to perform the tasks to the level of sham-OVX resilient controls. Akin to this finding, GCG-free HTP-GTE had no effect on the improvement of performance, and this further emphasizes the fundamental role of GCG as the major active substance in HTP-GTE underpinning the HTP-GTE-induced rescue of synaptic and cognitive impairments in postmenopausal depression.

## Discussion

LH is mediated by the failure to learn behavioral responses induced by inescapable aversive events. Hippocampus, the primary locus for learning and memory, has been known to play a major role in the development of LH where it is associated with its own functional and anatomical changes^38,60,61^. LH can occur with inescapable stress, which disrupts hippocampal LTP induction in vivo. However, cumulative evidences have raised questions regarding the role of the hippocampus in the development of depression. Passive response to inescapable shock is mediated by the serotonergic activity of the dorsal raphe nucleus (DRN), which results in escape failure. This passivity is overcome by learning control via the ventromedial prefrontal cortex (vmPFC), which inhibits DRN activity, making the rats possible to learn the fact that an escape from aversive stress is possible. Thus, the alterations in the vmPFC-DRN pathway serve as the main influence on the development of LH^62^. Consistent with these findings, we showed that both EGCG and GCG can effectively increase the resilience to escape the stress against foot shock-induced LH; however, only GCG can ameliorate hippocampal synaptic dysfunction and cognitive deficits induced by LH, and this difference between EGCG and GCG was clearly observed in Morris water maze tests in our results (Figs. 7 and S12). These results demonstrated that the failure of escaping behavior due to LH is mediated by a neural circuit, which is different from the hippocampal circuit responsible for the cognitive impairments by LH. Likewise, the administration of GCG is more effective in ameliorating depression-induced behavior than EGCG.

Although many studies have stressed the role of hippocampal synaptic plasticity as a possible mechanism underlying the cognitive impairment commonly accompanying depression^14–17^, a detailed synaptic and molecular mechanism remains to be elucidated. In the present study, we provided a possible mechanism to account for depression-induced cognitive deficits in terms of synaptic plasticity for the first time. The proportion of silent synapses containing NMDA receptor, but not AMPA receptor, in the hippocampus is significantly reduced in a PMD rodent model (Fig. 3), strongly implying a reduction in the actual formation of silent synapses in the cases of a depression-induced cognitive dysfunctions. In addition, the reemergence of silent synapses serves as the fundamentally mandatory mechanism for HTP-GTE-induced restoration of LTP in the hippocampus of a depressive animal model (Figs. 1 and 7).

BDNF plays a pivotal role in regulating a long-term synaptic plasticity in the hippocampus^48–50^, and the down-regulation of BDNF levels contributes to the depression-related cognitive impairments^5,51–53^. Concurrently, GluN2B-containing NMDA receptor plays a critical role in the formation and maintenance of silent synapses^58,59^. In fact, GluN2B signaling limits AMPA receptor incorporation in the developing synapses, resulting in the maintenance of a low AMPA/NMDA ratio at the immature glutamatergic synapses^59^. However, GluN2B deletion increases the number of functional synapses, which in turn prevents premature synapse maturation until correlated activity allows the induction of functional synapses^58^. Despite this circumstantial evidence indicating the involvement of BDNF in GluN2B-mediated regulation of silent synapses, the evidences to elucidate the relationship between BDNF and silent synapse formation has not yet been provided. In this study, we demonstrated that hippocampal BDNF expression is notably reduced in the helpless OVX rats, and that the suppressed BDNF-TrkB signaling pathway interferes with the hippocampal GluN2B expression, resulting in the reduced formation of silent synapses in the helpless OVX rats. Taken together, HTP-GTE rescues the dysfunctional long-term plasticity by reversing this process by increasing the hippocampal GluN2B expressions through the activation of BDNF-TrkB signaling pathway in the helpless OVX rats.

In conclusion, we developed a modified green tea extract, HTP-GTE, which is safer and more bioactive than conventional green tea extract. We demonstrated its therapeutic efficacy in overcoming postmenopausal depression as well as improving the cognitive dysfunctions associated with it. We also elucidated the potential mechanism underlying the effect of HTP-GTE by identifying GCG as a major bioactive component responsible for the effect. Our findings suggest that GCG-based green tea derivatives may serve as a potential therapeutic agent to prevent or ameliorate the cognitive deficits induced by postmenopausal depression.

## Materials and methods

### Animals

Female Sprague–Dawley rats (6-week-old, 140–160 g) were randomized by weight and housed in cages. They were housed three per cage and maintained with food and water on a 12-h light/dark cycle (lights on at 7 a.m. and lights off at 7 p.m.) in a temperature-controlled environment (23 ± 2 °C). The animals were initially distributed into two groups: placebo surgery (Sham) and ovariectomized (OVX) groups. On post-operative day 3, the OVX group was subdivided into three more groups which received vehicle (0.9% saline) or poly-phenolic compounds dissolved in vehicle (two different doses). Animal care was performed in accordance with the Yonsei University College of Medicine Animal Care (Project license number: #00,062; 2017–0070) and all experimental protocols were approved by Yonsei University College of Medicine and use Committee or the NIH Guide for the Care and Use of Laboratory Animals.

### Preparation of high temperature processed-green tea extract (HTP-GTE)

Fresh green tea (Camellia sinensis, CS) leaves were collected in spring from Osulloc Tea Garden in Jeju, Korea and were dried at 150 °C for 10 min. The dried CS leaf was extracted two times with 50% aqueous ethanol at 60 °C for 3 h. The 50% aqueous ethanol extract was decaffeinated by filtration with activated carbon and the incubated at high temperature for epimerization of catechins. The HTP-GTE was concentrated with a rotary evaporator (Buchi, Flawil, Switzerland) in vacuo and stored in a refrigerator (− 20 °C) prior to Ultra Performance Liquid Chromatography (UPLC) analysis.

### Preparation of GCG-free HTP-GTE

GCG-free HTP-GTE was prepared by the AmorePacific CO R&D center (Yongin, South Korea). GCG was isolated using a β-cyclodextrin-bonded silica column, and the amount of (−)-epigallocathechin-3-gallate (EGCG) was equalized to the conventional tea level by adding pure EGCG.

### UPLC-Photometric Diode Assay (UPLC-PDA) analysis

Bioactive components in HTP-GTE were determined by UPLC with a PDA detector using a Zorbax Eclipse XDB C18 column (2.1 mm × 100 mm, 1.8 μm; Agilent Technologies). The mobile phases were 0.05% Trifluoroacetic acid in water for solvent A and methanol/acetonitrile (7:3 (v/v) for solvent B. The mobile phase flow-rate was 1.0 mL/min and the injection volume was 2 μL.

### Preparation of human neuroblastoma SH-SY5Y cell and primary hippocampal neuron cultures

Primary hippocampal neuronal cell culture was produced and maintained using a method described previously^3^, with some modifications. All experiments were performed in accordance with the Yonsei University College of Medicine Animal Care and Use Committee or NIH Guide for the Care and Use of Laboratory Animals. Briefly, primary hippocampal neuron cultures were prepared from Sprague–Dawley rat embryos (embryonic day 18) of either sex. The hippocampi were dissected and the cells were dissociated by trituration using a fire-polished Pasteur pipette. The cells were then plated onto cover glasses coated with poly-L-lysine. The hippocampal cells were grown in neurobasal medium (Thermo Fisher Scientific, Waltham, CA, USA) supplemented with B-27 (2%) and L-glutamine (2 mM, Gibco, Waltham, MA, USA). The cultures were maintained at 37 °C in 5% CO_2_/95% air. Human neuroblastoma SH-SY5Y cells purchased from American Type Culture Collection (ATCC, Manassas, VA, USA) were maintained in Dulbecco’s Modified Eagle’s Medium (DMEM; Gibco, Waltham, MA, USA) supplemented with 10% fetal bovine serum (HyClone, GE healthcare, Chicago, IL, USA), 100 mg/mL streptomycin, and 100 U/mL penicillin (Gibco, Waltham, MA, USA) at 37 °C in a humidified incubator with 5% CO_2_.

### Surgical procedures of ovariectomy (OVX)

Six-week-old female rats were anesthetized with isoflurane (5% isoflurane, 95% O_2_) and bilateral ovariectomy was performed. The test group of female rats underwent ovariectomy (ovaries removed by surgical method) and the other group underwent sham surgery (the skin was cut and sewed back in place). The surgery consisted of a dorsolateral incision of the skin between the last rib and pelvis and muscle dissection to expose periovarian fat. Forceps were used to find the ovaries surrounded by variable amount of fat. The ovary was pulled out and the junction between the fallopian tube and uterine horn was cut. Bleeding was usually light and stopped soon. The horn and periovarian fat were returned into the abdominal cavity. The muscle wound was first sutured shut and the skin incision was closed. In sham surgery, the rats underwent the same incision but no ovaries were removed. To reduce pain, ketoprofen (2.5 mg/kg IM) was administered after surgery and the rats recovered within 3 days.

### Preparation of learned helplessness (LH) rat model by exposure to inescapable electric foot shock

The rats in the shock-exposed group received inescapable and unpredictable foot shock in an electric foot shock chamber (20.5 × 20 × 24 cm; Panlab, Barcelona, Spain). The shock chamber was equipped with metal rod (stainless steel) flooring connected to a shock generator and a shock control box (LE10026, LE900; Panlab, Barcelona, Spain). Inescapable foot shocks were delivered to rats repeatedly 50 times at an amplitude of 0.8 mA, a duration of 4 s, and randomized inter-shock intervals of 30–90 s over three consecutive days. Learned helplessness (LH) was assessed 24 h after the third shock procedure by testing escape performance, which comprised 30 escape trials. Each trial adopted a single 0.8 mA foot shock administered for a maximum duration of 15 s. For each escape trial, shock onset was accompanied by a sound/light cue that signaled door opening to permit escape into the adjacent compartment. Each trial was terminated when the rat escaped to the non-shock side of the shuttle box or the maximum duration (15 s) was reached. If the rat did not escape during shock, it was counted as a ‘‘failed’’ trial. Thirty shocks lasting for 15 s each were applied with an inter-trial time of 20 s. The rats with more than 20 escape failures in the 30 trials were regarded as being "helpless" and as having attained a state of learned helplessness^36,63^.

### Toxicity test

In vivo Toxicity test was carried out in compliance with the OECD Guidelines for the testing of chemicals, Acute Oral Toxicity – Fixed Dose Procedure (No. 420), and the Testing Guidelines for Safety Evaluation of Drugs (Notification No. 2009–19) issued by the Ministry of Food and Drug Safety. All rats were given a single oral dose of test articles after a 14-h fasting period. Doses (g/kg) were adjusted according to body weight recorded just before the examination. An initial dose volume of 10 mL/kg was used. A single dose of 5 g/kg was administered to animals. All visible signs of reaction to treatment and mortality were recorded daily. On the first day of treatment for each dose level, the animals were observed at the following approximate time points: at the end of dosing and 0.5, 1, 2, 3, 4, 5, and 6 h after dosing. Thereafter, any changes in clinical signs and/or mortality of test animals were observed daily during the test period. Then, body weights were recorded on the day of dosing, Day 1,4, 7, 10, 13, 16, 19, 22, 25 and 28. At the end of the observation period all animals were euthanized by a CO_2_ gas overdose. In terms of necropsy and gross pathology, any abnormalities were recorded, including details of location, color, shape, and size, and then appropriately sampled and identified.

### Cell viability test

In vitro toxicity test was done as follows. Cell viability was measured by the CCK-8 assay (Dojindo laboratories, Shanghai, China). SH-SY5Y cells were seeded at a density of 5 × 10^4^ cells per well in a 96-well plate and cultured until 70% confluence was attained. At 24-h post-treatment with vehicle [dimethyl sulfoxide (DMSO)], conventional green tea extract (GTE), or modified GCG-enriched green tea extract (HTP-GTE), the cells were incubated with 10 μL CCK-8 reagent for 2 h at 37 °C. The optical density was measured at a wavelength of 450 nm using a microplate reader (Molecular devices, San Jose, CA, USA). Cell viability was calculated by dividing the optical density of the treated group by that of the control group. Primary hippocampal neurons (2 × 10^6^ cells per well, 24-well plate) were cultured for 14 days to ensure complete maturation. At 2-h post-treatment with GTE or HTP-GTE, the medium was carefully replaced with fresh neurobasal medium containing dilute MTT (Sigma Aldrich, St. Louis, MO, USA) (1:10, 10% MTT) and incubated for another 2 h at 37 °C. After removing the incubation medium, the formazan crystals were dissolved in 200 μL DMSO. MTT reduction was quantified by measuring the light absorbance at 570 nm using the microplate reader^64^. The cell viability was calculated by dividing the optical density of the treated group by that of the control group.

### Hippocampal slice preparation

Hippocampal slices (400 μm thick) were prepared from learned helplessness (LH)-tested 11-week-old female Sprague Dawley rats. The rats were anesthetized with isoflurane (5% isoflurane, 95% O_2_) and perfused with ice-cold sucrose artificial cerebrospinal fluid (aCSF) with the following concentrations in mM: 195.5 sucrose, 2.5 KCl, 1 NaH_2_PO_4_, 32.5 NaHCO_3_, 11 glucose, 2 Na pyruvate, and 1 Na ascorbate (all chemicals from Sigma-Aldrich, St. Louis, MO, USA) bubbled with 95% O_2_/5% CO_2_ at a pH of 7.4. After perfusion, the brains were quickly removed from the skull and slices were cut on a vibratome (Leica biosystems, Wetzlar, Germany). The slices were transferred to an incubation chamber containing incubation solution with the following concentrations in mM: 119 NaCl, 2.5 KCl, 1 NaH_2_PO_4_, 26.2 NaHCO_3_, 11 glucose, 2 Na pyruvate, 1 Na ascorbate, 3 MgSO_4_, and 1.5 CaCl_2_ at 35 °C for 15 min. After incubation, the slices were transferred to a container filled with aCSF solution at 23—24 °C for 1 h.

### Electrophysiology

Field recordings were made with a concentric bipolar electrode positioned in the stratum radiatum of the CA1 region using an extracellular glass pipette (3—5 MΩ) filled with aCSF. Stimulation was delivered through a bipolar electrode (FHC, Bowdoin, ME, USA) placed in the Schaffer collateral-CA1 (SC). The SC circuit was visualized using differential interference contrast (DIC) microscopy at 4 × magnification and identified by the ability to evoke short and constant latency field excitatory postsynaptic potentials (fEPSPs) at CA1 synapses by SC input stimulation. The test stimulation in all fEPSP experiments was measured prior to the beginnging of all the experiments (30—300 μA) and a test-pulse stimulation strength that evoked 50% of the maximum fEPSP was used. Baseline synaptic responses were recorded for 30 min, and then long-term potentiation (LTP) was induced by high-frequency stimulation (HFS; 100 Hz, 4 trains, 1 s duration, 20 s inter-train interval). Recordings were made every 10 s for 1 h using Axopatch 1D amplifier (Molecular Devices, San Jose, CA, USA) digitized at 10 kHz, and filtered at 2 kHz with Digidata 1322A and pClamp 10.0 software (Molecular Devise, San Jose, CA, USA). For the minimal stimulation experiment, the lowest intensity was found to induce a combination of synaptic responses and failures by adjusting the SC stimulation intensity. Failure rate was calculated as the number of failures/total number of trials, and potency was calculated as the mean EPSC peak amplitude excluding failures^47,65^. The silent synapse percent was measured using the following equation: 1-Ln(F_-70_)/Ln(F_+40_) (F_-70_: failure rate at -70 mV, F_+40_: failure rate at + 40 mV). The criterion of single axon stimulation was co-initiation or no response, and the average EPSC amplitude remained unchanged because of the small increase in stimulus intensity^4,5^. To measure miniature EPSCs, the electrode was filled with internal solution (concentration in mM: 135 Cs methanesulfonate, 8 NaCl, 10 HEPES, 0.5 EGTA, 4 Mg-ATP, 0.3 Na-GTP, and 5 QX-315 Cl; pH 7.25 with CsOH, 285 mOsm). Miniature currents were recorded in the presence of tetrodotoxin (1 μM TTX, Tocris, Bristol, England) to block sodium currents and propagate action potentials. Spontaneous firing was recorded in the cell attached mode from CA3 principal cells and stratum radiatum interneurons.

### Preparation of synaptoneurosome fraction

Total lysate and synaptoneurosome (SN) were prepared from the hippocampus. The isolated hippocampus was homogenized with 0.32 M sucrose in 4 mM HEPES solution. The lysates were then centrifuged at 1000 × g for 10 min at 4 °C. The supernatant was transferred to the new collection tube and centrifuged at 10,000 × g for 15 min at 4 °C. The resulting supernatant (total lysate) was stored, and the remaining pellets were re-suspended with 0.32 M HEPES buffered sucrose solution. The re-suspended sample was centrifuged at 10,000 × g for 15 min at 4 °C. To lyse the sample, the pellets were re-suspended in dd H_2_O in order to provide a hypo-osmotic shock, transferred to a glass–Teflon tissue homogenizer, and homogenized rapidly by hand (3 strokes). The sample was transferred to a new collection tube, suspended in 4 mM HEPES solution prepared by the addition of 1 M HEPES, and rotated for 30 min at 4 °C to ensure complete lysis. The lysed sample was centrifuged at 25,000 × g for 20 min at 4 °C, and then the supernatant was removed. Pellets were re-suspended in 0.32 M HEPES buffered sucrose solution (synaptoneurosome fraction) and subjected to western blotting.

### Western blotting

The rats were anesthetized with isoflurane (5% isoflurane, 95% O_2_) and perfused with ice-cold sucrose artificial cerebrospinal fluid (aCSF) solution. From the rat brains, the hippocampus was dissected and samples were homogenized for 10 min with lysis buffer (1% Triton X-100, 0.32 M sucrose in HEPES solution) on ice with Halt protease/phosphatase inhibitor cocktail (Thermo Fisher Scientific, Waltham, CA, USA). Protein concentration was determined using a BCA Protein Assay Kit (Thermo Fisher Scientific, Waltham, CA, USA). Equal amounts of protein were loaded onto a 12% sodium dodecyl sulfate (SDS)-polyacrylamide gel for separation and then transferred onto 0.45-μm size pore polyvinylidene fluoride membranes. The membranes were blocked with 5% nonfat dry milk plus 0.1% Tween 20 for 2 h, and protein levels were analyzed by immunoblotting with primary antibodies, including GluN1, GluN2A, GluN2B (Sigma Aldrich, St. Louis, MO, USA), BDNF (Alomone, Jerusalem, Israel), and β-actin for normalization (SCBT, Dallas, TX, USA). All full blots are represented in Figs. S13, S14.

### RNA extraction and quantitative real-time PCR

Total RNA was extracted from tissue using RNase mini kit (Qiagen, Hilden, Germany) according to the manufacturer’s instructions. The Superscript III First-strand synthesis system for RT-PCR kit (Invitrogen, Carlsbad, CA, USA) was used for the Real-Time PCR analysis to see the expression levels of BDNF and GluN2B. The primers were as follows: BDNF forward: 5′-TGGCTGACACTTTTGAGCAC-3′; reverse: 5′-TTTTCTTCGTTGGGCCGAAC-3′, and GluN2B forward: 5′-GCTTCTACCGGCTAT GGCAT-3′; reverse: CTGCGACGATGCTCAGAGAT-3′ and GAPDH forward: 5′-GAAGGTCG GTGTGAACGGAT-3′; reverse: AGTGATGGCATGGACTGTGG-3′. The reaction carried out in a system of 10 μl, containing 50 ng cDNA templates, specific primers (2.5 μM of primer mix), 5 μl SYBR green master mix (Applied Biosystems, 2 ×), and RNase Free water. The amplification procedures were performed in Applied Biosystems 7300 real-time PCR system (Applied Biosystems, Foster City, CA, USA). The expression levels of the target genes were calculated using (2^-ΔΔCt^) method.

### Morris water maze (MWM) test

The rats were trained in the water maze to find a hidden platform for 6 consecutive days. On the first day, the trial began by placing the rats into the tank facing the mark on the wall at one of the four quadrants and ended when the rats climbed on the platform. If the rats were not able to find the platform within 1 min, they were guided to the platform and allowed to stay on the platform for 10 s only once. From the second day to the fifth day, the trial began by placing the rats into the tank facing the same mark as in the first day trial and were evaluated for their ability to find the platform in 2 min. Four trials were conducted per day (with a 20-min interval). On the sixth day of the test, the hidden platform was removed. The percentage of time spent in the target quadrant and the platform crossing number were recorded using a video camera and tracking data were visualized by EthoVision XT software (Noldus, Wageningen, Netherlands).

### Golgi staining and morphological analysis of dendritic spines

Dendritic spine density and morphology analysis in the hippocampus were performed using the FD Rapid GolgiStain Kit (FD NeuroTechnologies, Columbia, MD, USA) according to the manufacturer’s protocol. In brief, the brain was immersed in 10 mL of a mixture containing equal amounts of solution A and B and stored at 23—24 °C for 2 weeks with gentle agitation. The brain was then transferred to solution C, stored at 4 °C for 48 h, and then frozen in optimal cutting temperature (OCT) compound at -20 °C for 12 h. Brain sections with 150-μm thickness were dissected with dry ice using a cryostat microtome. Each section was mounted onto a slide and allowed to dry naturally at room temperature. In the next step, the slide was stained with a solution (a mixture of solution D, E, and distilled water in a 1:1:2 ratio) for 5—10 min, and washed with distilled water twice for 5 min each. The slides were dehydrated with 50%, 75%, 80%, 90%, and 100% ethanol sequentially, and cleared with xylene. Finally, the slides were visualized using a Zeiss LSM710 confocal microscope (Carl Zeiss, Jena, Germany). Confocal z-series image stacks encompassing entire dendrite segments were analyzed using MetaMorph software (Molecular Devices, San Jose, CA, USA). To measure dendritic spine density, we evaluated the dendritic segments 50 μm from the first branch of main axon from soma of the hippocampus CA1. Spines 0.2—2 μm long were included for analysis (n = 3 brains/group).

### Rotarod test

Rotarod tests were performed to compare motor dysfunction before and after ovariectomy or electrical shock, animals were tested on the rotarod (B.S Technolab, Daejeon, Korea). Before ovariectomy, they had time for adaptive training sessions first (5 RPM, 5 min) and three consecutive sessions under an accelerating speed (5–40 RPM, 300 s). The day after electric shock for learned helpless test, rotarod performance was evaluated with three sessions under accelerated speed as described above. Each group were exposed to the rod three times for training before taking the test. For each of the test, results are expressed as the mean latency to fall for trials.

### Serum 17β-Estradiol ELISA assay

The 17β-estradiol levels were detected by chemilluminescence. An estradiol ELISA kit (Enzo life sciences, Farmingdale, NY, USA) was used to detect the level of corresponding substances. The detailed steps were carried out according to the manufacturer’s protocol. The results were detected with Multiskan sky microplate spectrophotometer (Thermo Fisher Scientific, Waltham, MA, USA).

### Statistics

All data are reported as mean ± SEM. Statistical comparisons were made using the two-tail unpaired *t* test to compare the two independent group means. Means for more than three independent groups were compared using the one-way analysis of variance (ANOVA) followed by Tukey’s post hoc test for the multiple comparisons. The two-way repeated-measures (RM) ANOVA was used to compare the dependent group means across the Morris water maze training sessions, which was followed by Tukey’s post hoc test for the multiple comparisons (Fig. 7j). Kolmogorov–Smirnov two-sample test was used to compare the cumulative distributions of two data sets (Figs. 2, S7, S11). Data were analyzed using Prism software (GraphPad, La Jolla, CA, USA). Differences were considered significant when p < 0.05.

## Supplementary Information

Supplementary Information.

## Data Availability

All data generated or analysed during this study are included in this published article (and its Raw data zip file).
