# Supplementary material for: (−)-Gallocatechin gallate from green tea rescues cognitive impairment through restoring hippocampal silent synapses in post-menopausal depression
Source: Sci Rep. 2021 Jan 13;11:910. doi: 10.1038/s41598-020-79287-x (PMC7806886; doi:10.1038/s41598-020-79287-x)
Supplement: Supplementary file 1 — Supplementary Information. [file 41598_2020_79287_MOESM1_ESM.docx]

**Supplementary information**

**(-)-gallocatechin gallate from green tea rescues cognitive impairment through restoring hippocampal silent synapses in post-menopausal depression.**

Sukjin Ko,^1,^†Won Seuk Jang,^2,^†Ji-Hyun Jeong,^1^ Ji Woong Ahn,^1^ Young-Hwan Kim,^3^ Sohyun Kim,^1,^‡, Hyung kyung Chae,^3^ Seungsoo Chung^1,*^

Fig. S1. Characteristics of HTP-GTE on toxicology and neuronal cell viability.

Fig. S2. HTP-GTE administration improves resilience in OVX rats.

Fig. S3. HTP-GTE does not affect hippocampal synaptic functions in shock-free Sham or OVX rats.

Fig. S4. Effects of HTP-GTE on Locomotor activity and 17β-estradiol hormone level.

Fig. S5. LH-induced synaptic impairments at Schaffer collateral-CA1 synapses in hippocampus.

Fig. S6. HTP-GTE rescues the synaptic impairments by restoring LTP in OVX.

Fig. S7. LH-induced synaptic impairment at hippocampal circuit in female rats.

Fig. S8. LH-induced impairment of silent synapses in female rats.

Fig. S9. Silent synapses are reemerged following HTP-GTE administration in OVX rats.

Fig. S10. BDNF level in hippocampus following HTP-GTE administration in OVX rats.

Fig. S11. Regulation of BDNF-TrkB pathway underlies HTP-GTE-dependent amelioration of hippocampal synaptic impairment in helpless OVX rats.

Fig. S12. GCG, but not EGCG, plays a role as a major active component in HTP-GTE -induced improvements of synaptic and cognitive impairments in the helpless OVX rats.

Fig. S13. Full-length blot of figures

Fig. S14. Full-length blot of figures

Table S1. Composition of catechins and caffeine of GTE and HTP-GTE.


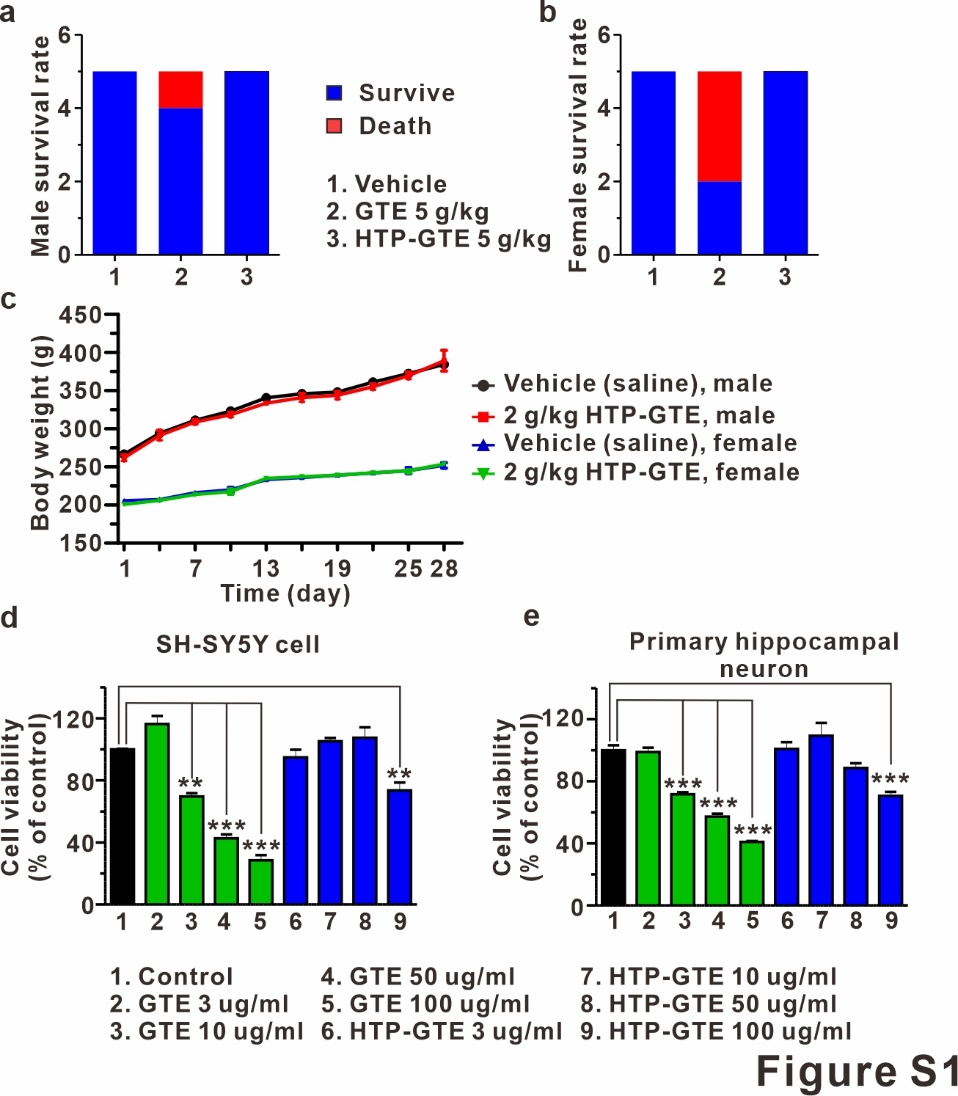


**Fig. S1. Characteristics of HTP-GTE on toxicology and neuronal cell viability.**

**a, b**, Survival rate from GTE and HTP-GTE administration in male and female rats (Vehicle n = 5, GTE n = 5, HTP-GTE n = 5). **c**, Body weight changes from male and female rats (Vehicle male, Vehicle female, HTP-GTE male, and HTP-GTE female, n = 5). **d**, Cell viability in human SH-SY5Y cells (Control: 100$\pm$ 0.32; GTE 3 μg/ml: 116.3 $\pm$ 5.15; GTE 10 μg/ml: 69.69 $\pm$ 2.11; GTE 50 μg/ml: 42.75 $\pm$ 2.44; GTE 100 μg/ml: 28.58 $\pm$ 3.23; HTP-GTE 3 μg/ml: 94.85 $\pm$ 4.88; HTP-GTE 10 μg/ml: 105 $\pm$ 2.15; HTP-GTE 50 μg/ml: 107.4 $\pm$ 6.74; HTP-GTE 100 μg/ml: 73.56 $\pm$ 5.11; n = 3). **e**, Cell viability in primary hippocampal neurons (Control: 100$\pm$ 3.21; GTE 3 μg/ml: 98.85 $\pm$ 2.78; GTE 10 μg/ml: 71.6 $\pm$ 1.36; GTE 50 μg/ml: 57.19 $\pm$ 1.89; GTE 100 μg/ml: 40.94 $\pm$ 0.61; HTP-GTE 3 μg/ml: 100.9 $\pm$ 4.35; HTP-GTE 10 μg/ml: 109.4 $\pm$ 8.20; HTP-GTE 50 μg/ml: 88.59 $\pm$ 3.07; HTP-GTE 100 μg/ml: 70.6 $\pm$ 2.57; n = 6). Data are represented as mean $\pm$ SEM (One-way ANOVA Tukey’s *post hoc* test, **p < 0.01, ***p < 0.001).


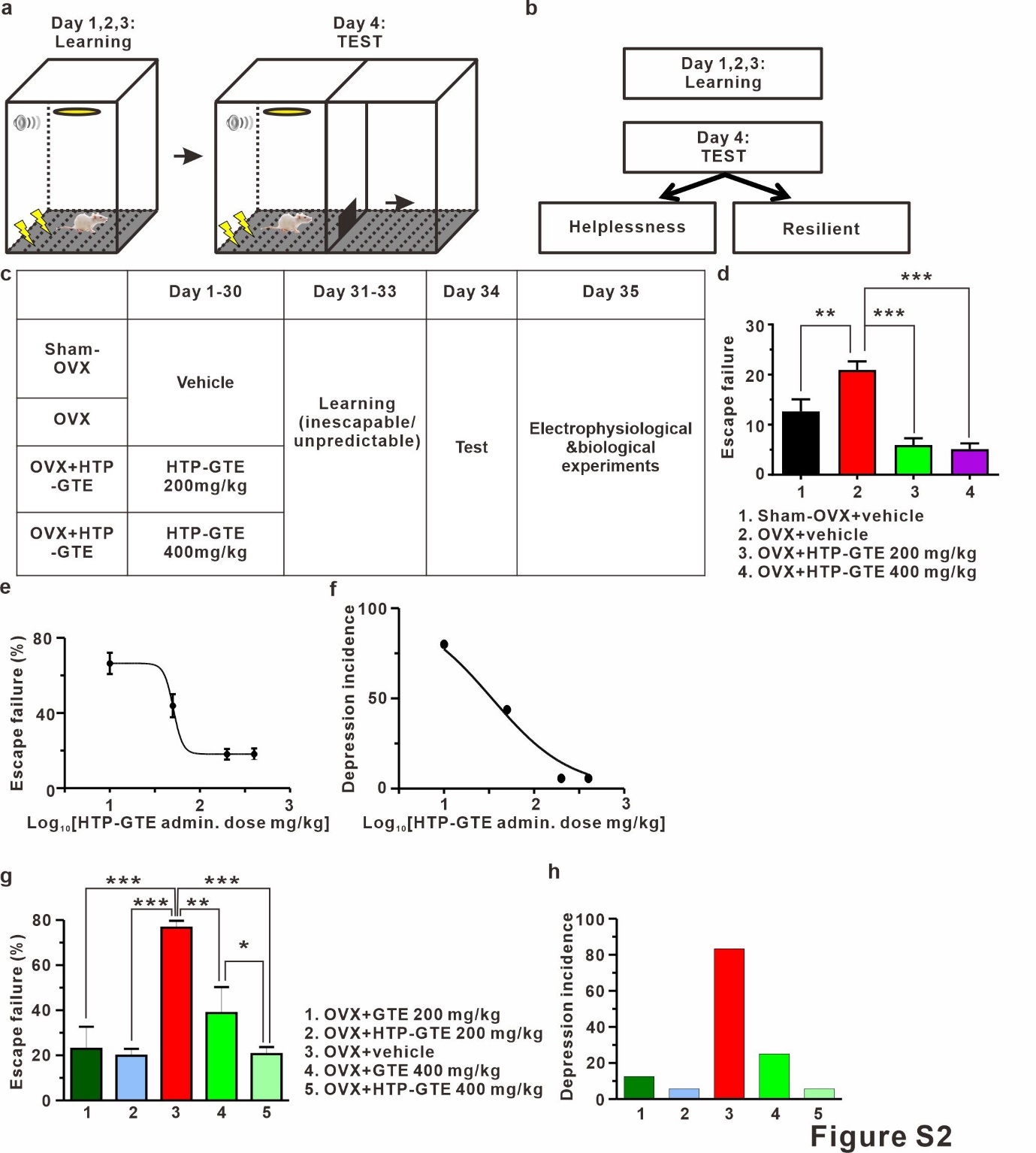


**Fig. S2. HTP-GTE administration improves resilience in OVX rats.**

**a-c**, Learned helplessness protocol and time schedule. **d,** Summary of escape failure in Sham-OVX+vehicle, OVX+vehicle, OVX+HTP-GTE (200 mg/kg), and OVX+HTP-GTE (400 mg/kg) groups (Sham-OVX+vehicle: 12.6 ± 2.4, n = 19; OVX+vehicle: 21.0 ± 1.7, n = 19; OVX+HTP-GTE 200 mg/kg: 5.9 ± 1.3, n = 19; OVX+HTP-GTE 400 mg/kg: 5.1 ± 1.2, n = 19). **e-f**, Dose-response relationships for the escape failure and depression incidence for OVX+HTP-GTE ±group. **g-h,** summary of the escape failure and depression incidence in OVX+vehicle, OVX+GTE, OVX+HTP-GTE groups (Escape failure; OVX+vehicle: 76.61$\pm$ 3.07, n = 42; OVX+GTE 200 mg/kg: 22.92$\pm$ 9.79, n = 8; OVX+HTP-GTE 200 mg/kg: 19.81 $\pm$ 3.12, n = 35; OVX+GTE 400 mg/kg: 38.75 $\pm$ 11.53, n = 8; OVX+HTP-GTE 400 mg/kg: 20.57 $\pm$ 3.12, n = 35). Data are represented as mean $\pm$ SEM (One-way ANOVA Tukey’s *post hoc* test, *p < 0.05, **p < 0.01, ***p < 0.001).


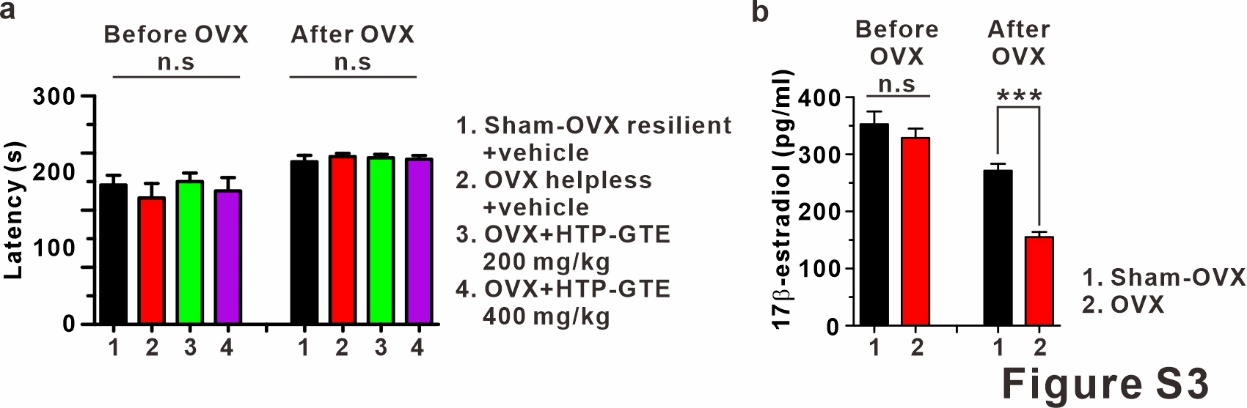


**Fig. S3. Effects of HTP-GTE on Locomotor activity and 17β-estradiol hormone level.**

**a,** comparison of the locomotor activity in each group (before: Sham OVX resilient+vehicle: 244 $\pm$ 17.1; OVX helpless+vehicle: 222 $\pm$ 25.4; OVX+HTP-GTE 200 mg/kg: 250 $\pm$ 15.1; OVX+HTP-GTE 400 mg/kg: 234 $\pm$ 23.1, after: Sham OVX resilient+vehicle: 285 $\pm$ 11.5; OVX helpless+vehicle: 294 $\pm$ 5.69; OVX+HTP-GTE 200 mg/kg: 292 $\pm$ 6.06; OVX+HTP-GTE 400 mg/kg: 289 $\pm$ 6.69; n=10/group). **b**, comparison of 17β-estradiol hormone level in each group (pg/ml, before: Sham OVX resilient+vehicle: 353 $\pm$ 22.1; OVX helpless+vehicle: 329 $\pm$ 16.3; OVX+HTP-GTE 200 mg/kg: 331 $\pm$ 15.7; OVX+HTP-GTE 400 mg/kg: 301 $\pm$ 13.8, after: Sham OVX resilient+vehicle: 271 $\pm$ 12.2; OVX helpless+vehicle: 155$\pm$ 8.94; OVX+HTP-GTE 200 mg/kg: 286$\pm$ 29.6; OVX+HTP-GTE 400 mg/kg: 284$\pm$ 16.2; n=5/group). Data are represented as mean $\pm$ SEM (One-way ANOVA/Tukey’s post hoc test, ***p < 0.001).


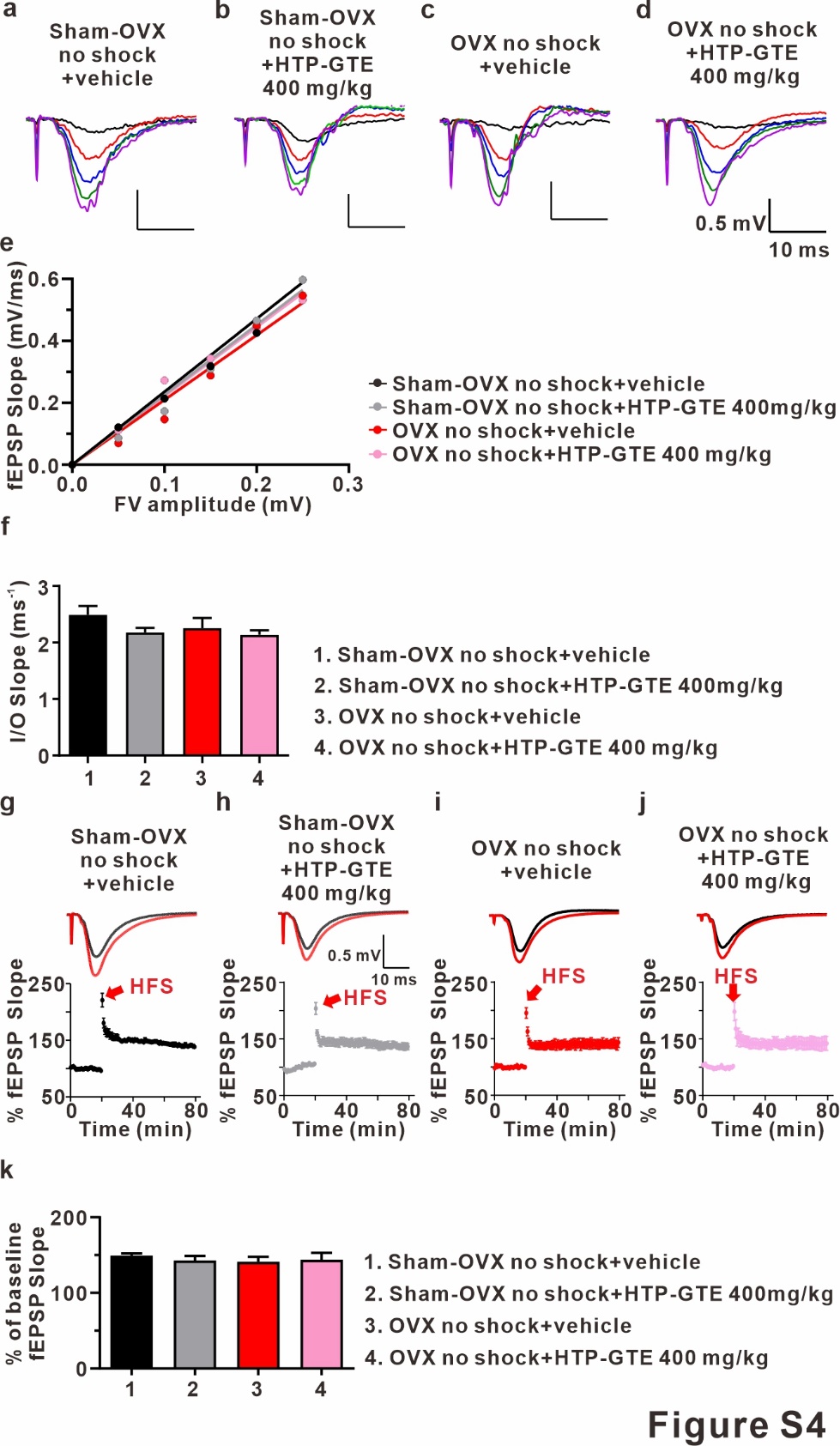


**Fig. S4. HTP-GTE does not affect hippocampal synaptic functions in shock-free Sham or OVX rats.**

**a-d**, Representative traces of fEPSPs from hippocampal slices from representative experiments at four increasing stimulus intensities in Sham on shock+vehicle, Sham no shokc+HTP+GTE 400 mg/kg, OVX no shock+vehicle, and OVX no shock+HTP-GTE 400 mg/kg groups **e**, The scatter plot of the Input and Output (I/O) relationship corresponding to the recorded fEPSPs in a. **f**, The average of slope I/O relationship in each group (Sham no shock+vehicle: 2.49$\pm$0.16, n = 7 slices/3 rats; Sham no shock+HTP-GTE 200 mg/kg: 2.18$\pm$0.08, n = 7 slices/3 rats; OVX no shock+vehicle: 2.56 $\pm$ 0.18, n = 8 slices/3 rats; OVX no shock+HTP-GTE 200 mg/kg: 2.14 $\pm$ 0.08, n = 7 slices/3 rats). **g-j**, Top: representative traces showing field EPSPs before (average of 20 traces, black line) and after (average of 180 traces, red line) high-frequency stimulus in each group. Bottom: average time courses for field EPSP amplitude during LTP induction in each group. Data are shown as mean $\pm$SEM. **k**, Summary of LTP induction in each groups (Sham no shock+vehicle: 149$\pm$2.72, n = 7 slices/3 rats; Sham no shock+HTP-GTE 400 mg/kg: 143$\pm$5.97, n = 7 slices/3 rats; OVX no shock+vehicle: 141 $\pm$ 6.9, n = 8 slices/3 rats; OVX no shock + HTP-GTE 400 mg/kg: 144 $\pm$ 9.14, n = 7 slices/3 rats).


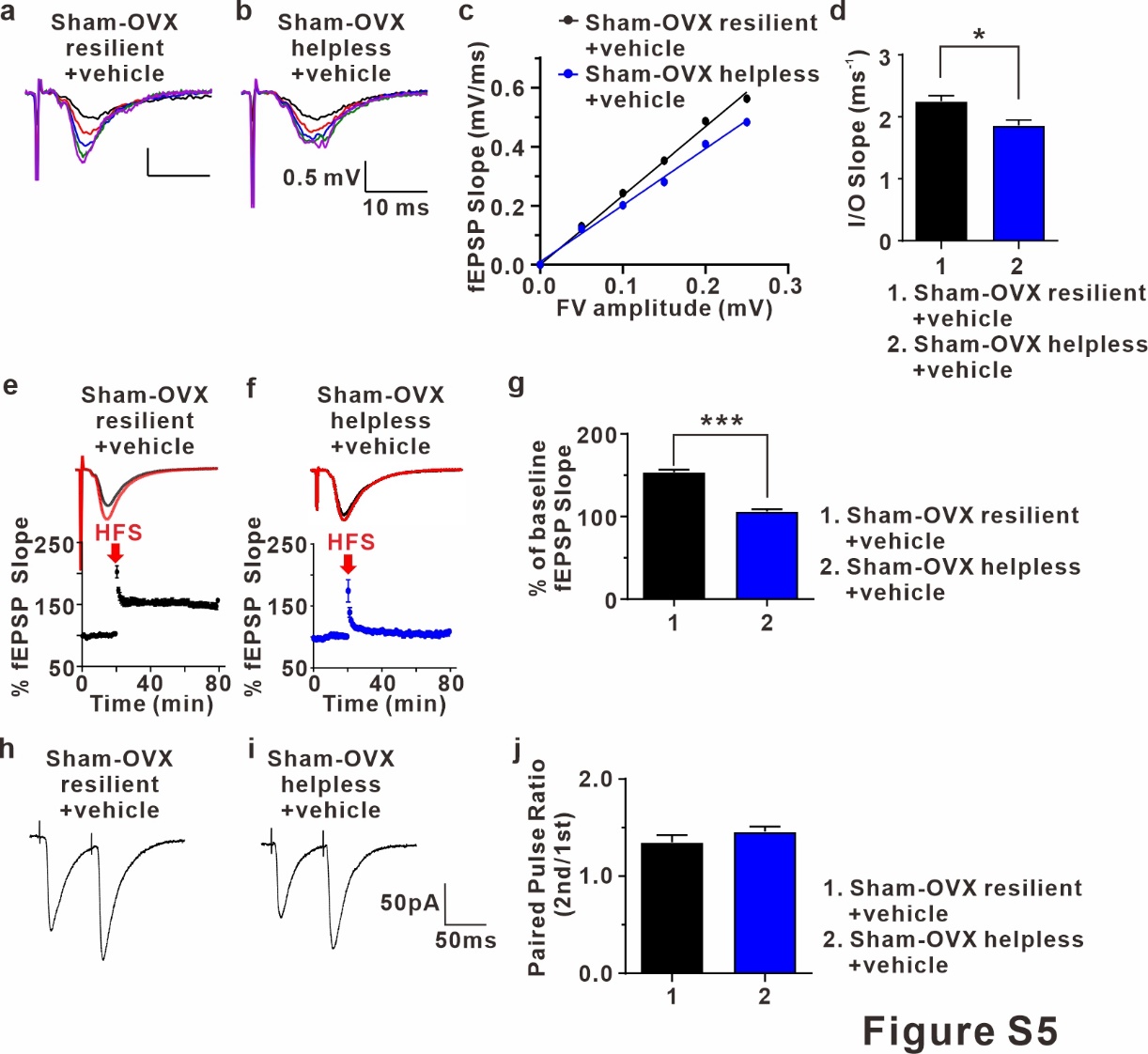


**Fig. S5. LH-induced synaptic impairments at Schaffer collateral-CA1 synapses in hippocampus.**

**a-b**, Representative traces of fEPSPs from hippocampal slices from sham-OVX resilient+vehicle and sham OVX helpless+vehicle groups at four increasing stimulus intensities. **c**, The scatter plot of the Input and Output (I/O) relationship corresponding to the recorded fEPSPs in a. **d**, The average of slope I/O relationship for sham resilient and sham helpless (Sham-OVX resilient+vehicle: 2.25$\pm$0.09, n = 6 slices/3 rats; Sham-OVX helpless+vehicle: 1.86 $\pm$0.09, n = 6 slices/3 rats). **e-f**, Top: representative traces showing field EPSPs before (average of 20 traces, black line) and after (average of 180 traces, red line) high-frequency stimulus in sham-OVX resilient+vehicle and sham-OVX helpless+vehicle groups. Bottom: average time courses for field EPSP amplitude during LTP induction in each group. Data are shown as mean $\pm$SEM. **g**, summary of LTP induction in each group (Sham-OVX resilient+vehicle: 153.3 $\pm$ 3.39, n = 5 slices/3 rats; Sham-OVX helpless+vehicle: 106 $\pm$ 2.69, n = 6 slices/3 rats). **h-i**, Representative traces of paired pulse-stimulation evoked EPSCs (50 Hz; average of 10 trials) in each group. **j**, summary of paired pulse ratio (PPR) of evoked EPSCS in each group (Sham-OVX resilient+vehicle: 1.35 $\pm$ 0.07, n = 6 cells/3 rats; Sham-OVX helpless+vehicle: 1.46 $\pm$ 0.05, n = 6 cells/3 rats n = 8 cells/4 rats). Data are represented as mean $\pm$ SEM (Unpaired student’s t-test, *p < 0.05, **p < 0.01, ***p < 0.001).


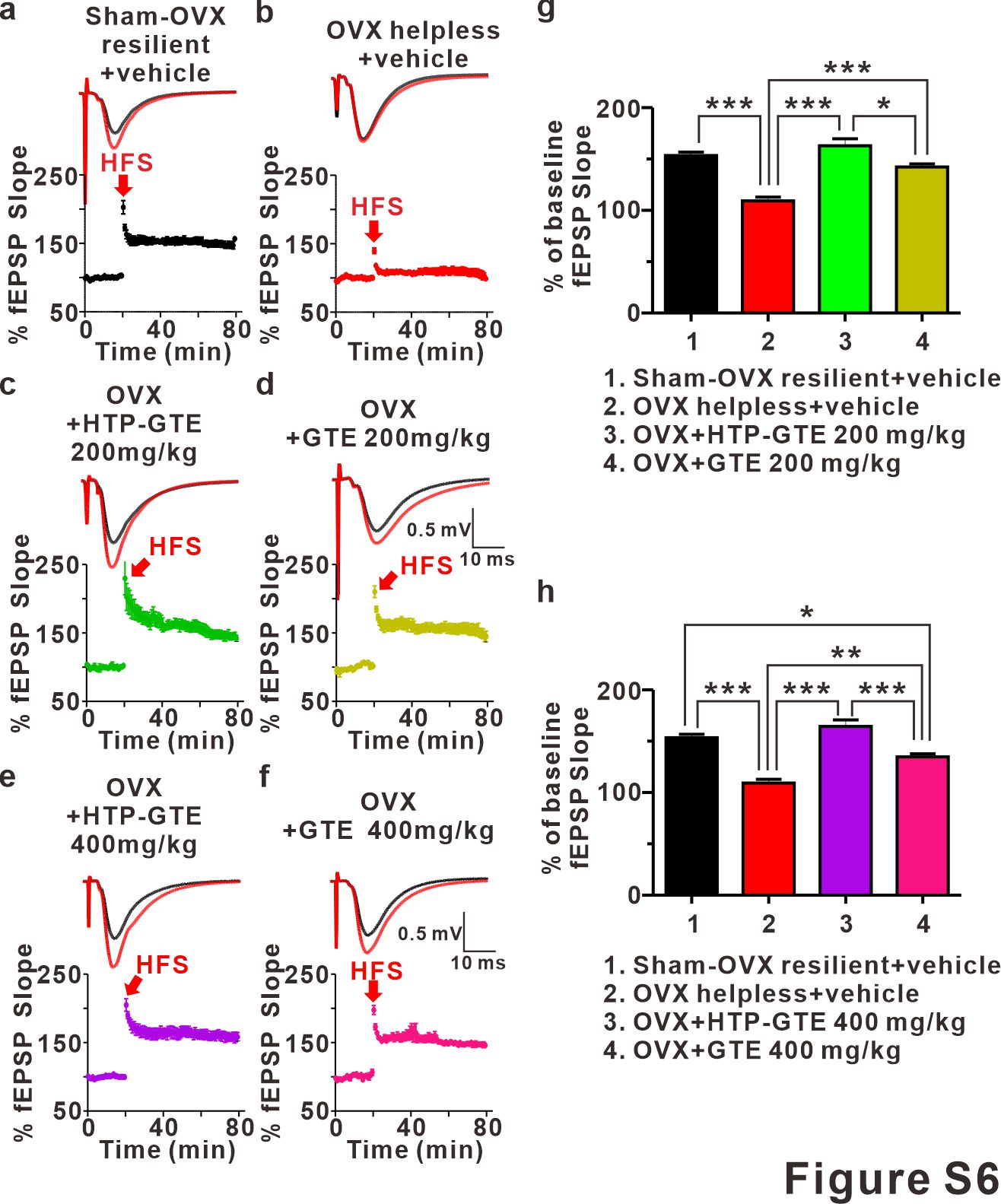


**Fig. S6. HTP-GTE rescues the synaptic impairments by restoring LTP in OVX.**

**a-f**, Top: representative traces showing EPSPs before (average of 20 traces, black line) and after (average of 180 traces, red line) high-frequency stimulus in Sham-OVX resilient+vehicle, OVX helpless+vehicle, OVX+HTP-GTE 200 mg/kg, OVX+GTE 200 mg/kg, OVX+HTP-GTE 400 mg/kg, and OVX+GTE 400 mg/kg groups. Bottom: average time courses for EPSP amplitude during LTP induction in each group. **g-h**, Summary of the LTP induction in each groups (Sham-OVX resilient+vehicle: 153.3 $\pm$ 3.39, n = 5 slices/3 rats; OVX helpless+vehicle: 109.2 $\pm$ 3.65, n = 6 slices/3 rats; OVX+HTP-GTE 200 mg/kg: 162.9$\pm$ 6.81, n = 5 slices/3 rats; OVX+GTE 200 mg/kg: 143.1$\pm$5.48, n = 5 slices/3 rats OVX+HTP-GTE 400 mg/kg: 164.5$\pm$ 6.27, n = 5 slices/3 rats; OVX+GTE 400 mg/kg: 135.7$\pm$3.65, n = 6 slices/3 rats). Data are represented as mean $\pm$ SEM (One-way ANOVA/Tukey’s *post hoc* test, *p < 0.05, **p < 0.01, ***p < 0.001).


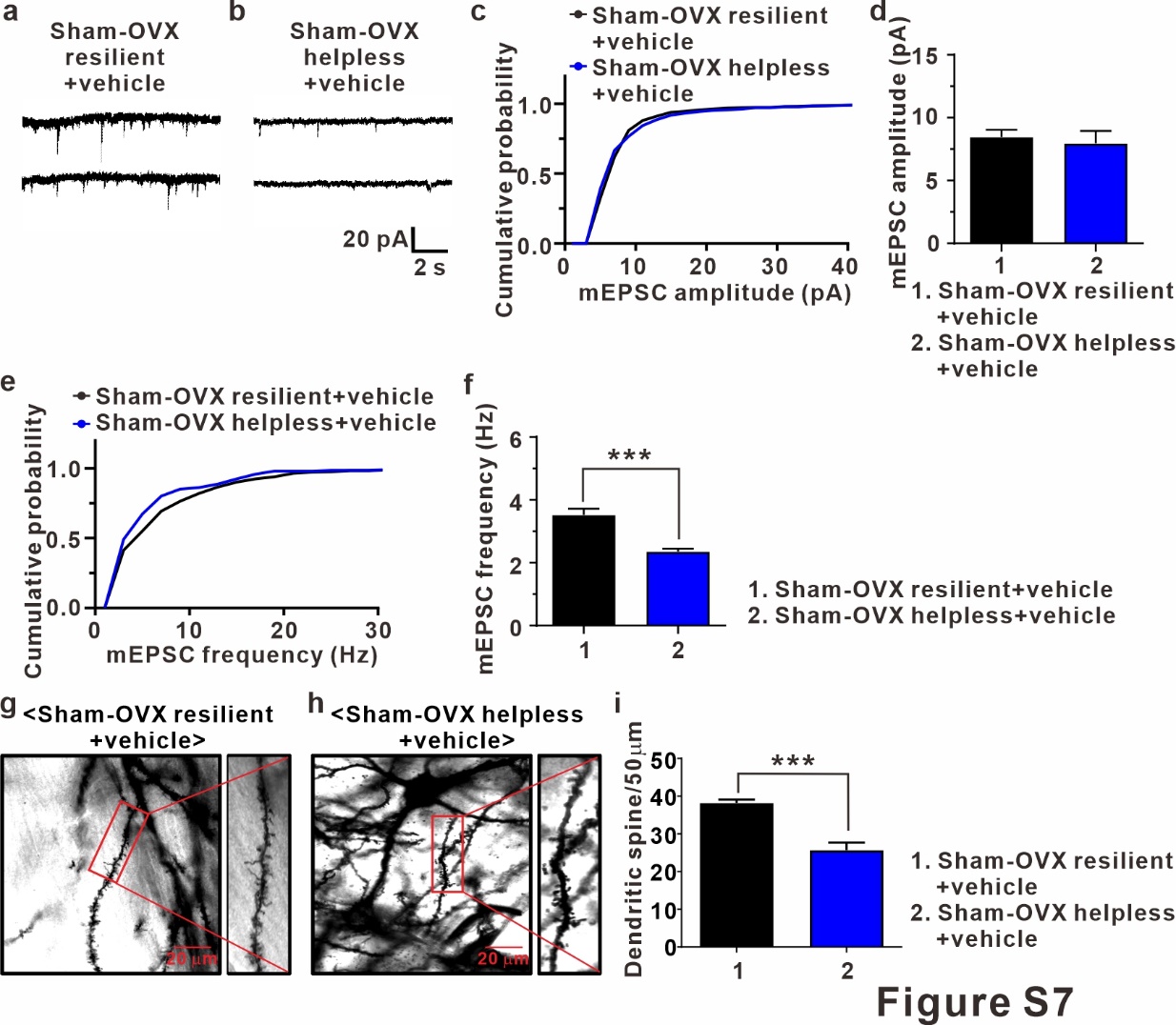


**Fig. S7. LH-induced synaptic impairment at hippocampal circuit in female rats.**

**a-b**, Sample traces showed miniature EPSCs in sham-OVX resilient+vehicle and Sham-OVX helpless+vehicle groups. **c**, Cumulative probability plot of mEPSC amplitudes for both groups. (Sham-OVX resilient+vehicle vs sham-OVX helpless+vehicle: p=0.9952, Kolmogorov-Smirnov two-sample test). **d**, Mean amplitude of mEPSCs for both groups (Sham-OVX resilient+vehicle: 8.47 $\pm$ 0.57, n = 6 slices/3 rats; Sham-OVX helpless+vehicle: 7.95 $\pm$ 0.99, n = 7 slices/3 rats). **e**, Cumulative probability plot of mEPSC frequencies for both groups. (Sham-OVX resilient+vehicle vs Sham-OVX helpless+vehicle: p=0.872, Kolmogorov-Smirnov two-sample test). **f**, Mean frequency of mEPSCs for both gbroups (Sham-OVX resilient+vehicle: 3.52 $\pm$ 0.19, n = 6 slices/3 rats; Sham helpless: 2.36 $\pm$ 0.09, n = 7 slices/3 rats). **g-h**, Representative golgi-stained dendritic segments of CA1 pyramidal neuron from both groups. **i**, Quantitative analysis of spine density for both groups (Sham-OVX resilient+vehicle: 38.2 $\pm$ 0.91; Sham-OVX helpless+vehicle: 25.7 $\pm$ 1.96; 20 segments/3-4 brains/group). Data are represented as mean $\pm$ SEM (Unpaired student’s t-test, *p < 0.05, **p < 0.01, ***p < 0.001).


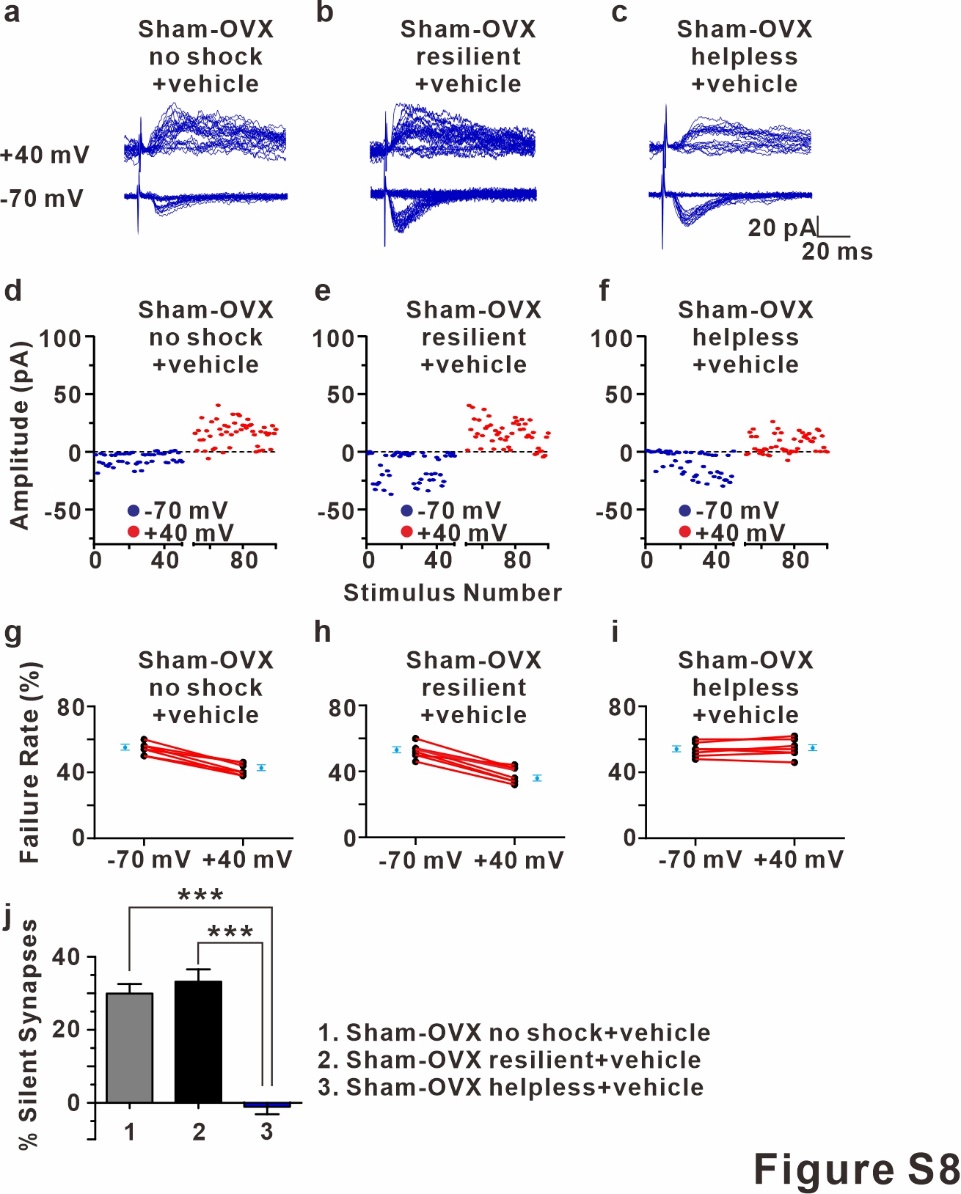


**Fig. S8. LH-induced impairment of silent synapses in female rats.**

**a-c**, Representative traces for EPSCs evoked by minimal stimulation for 50 trials at holding potentials of -70 mV or +40 mV in the hippocampal slices from sham-OVX no shock+vehicle, sham-OVX resilient+vehicle, and sham-OVX helpless+vehicle groups. **d-f**, Time course of EPSC amplitudes corresponding to the recorded EPSCs in **a**. **g-i**, Failure rates for EPSCs at -70 mV or +40 mV in the hippocampal slices for all groups. **g**, Percentage of silent synapse proportions for all groups (Sham-OVX no shock: 29.9 $\pm$ 2.18, n = 7 Sham-OVX resilient: 33.2 $\pm$ 3.38, n = 7; Sham helpless: 3.93 $\pm$ 3.89, n = 6). Data are represented as means $\pm$ SEM (One-way ANOVA Tukey’s post hoc test, ***p < 0.001).


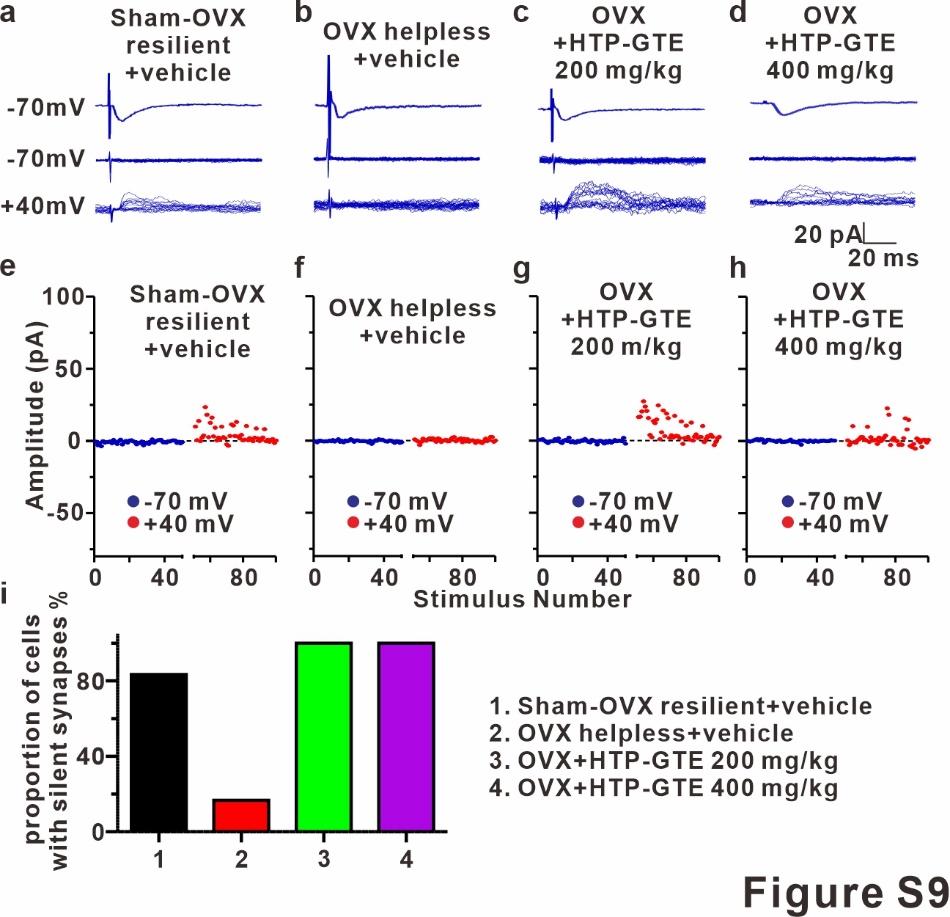


**Fig. S9. Silent synapses are reemerged following HTP-GTE administration in OVX rats**

**a-d**, 1st traces (from top to bottom), representative traces for AMPA EPSCs evoked by minimal stimulation in the hippocampal slices from sham-OVX resilient+vehicle, OVX helpless+vehicle, OVX+HTP-GTE 200 mg/kg, and OVX+HTP-GTE 400 mg/kg groups. 2nd traces, representative traces for failure of EPSC triggering when the stimulus intensity was reduced to maximal sub-threshold level for triggering EPSCs. Maximal sub-threshold intensities were determined as a maximal stimulus intensity at which EPSCs triggering failed as stimulus intensity was reduced to sub-threshold level by 1 μA steps. 3rd traces, representative traces for NMDA-only EPSCs at isolated silent synapses at SC inputs in all groups. NMDA EPSCs at isolated silent synapses were triggered by maximal sub-threshold stimuli in Sham-OVX resilient+vehicle, OVX+HTP-GTE treated groups contrast to OVX helpless+vehicle group. **e-h**, Time-courses for AMPA and NMDA EPSCs recorded in **a-d**. **i**, Proportion of cells expressing NMDAR-only synapses in all groups (Sham-OVX resilient+vehicle, n = 8; OVX helpless+vehicle, n = 9; OVX+ HTP-GTE 200 mg/kg, n = 8; OVX+ HTP-GTE 400 mg/kg, n = 8).Data are represented as means $\pm$ SEM (One-way ANOVA Tukey’s post hoc test, ***p < 0.001).


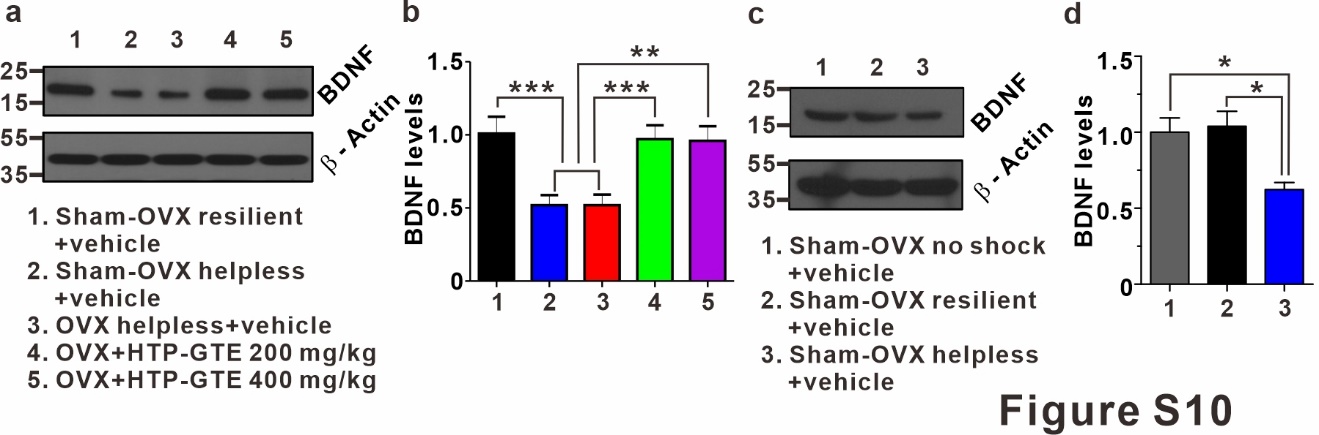


**Fig. S10. BDNF level in hippocampus following HTP-GTE administration in OVX rats.**

**a-b**, Western blot of BDNF level in hippocampus on sham-OVX resilient+vehicle, sham-OVX helpless+vehicle, OVX helpless+vehicle, OVX+HTP-GTE 200 mg/kg, and OVX+HTP-GTE 400 mg/kg groups in oral administration of HTP-GTE or vehicle solution daily for 4 weeks (Sham-OVX resilient+vehicle: 1.00 $\pm$ 0.06; Sham-OVX helpless+vehicle: 0.52 $\pm$ 0.04; OVX helpless+vehicle: 0.52 $\pm$ 0.04; OVX+HTP-GTE 200 mg/kg: 0.97 $\pm$ 0.06; OVX+HTP-GTE 400 mg/kg: 0.96 $\pm$ 0.06, n = 3 brains/group). **c-d**, Western blot of BDNF level in the hippocampus of each group in oral administration of vehicle solution daily for 4 weeks (Sham-OVX no shock+vehicle: 1.00 $\pm$ 0.09; Sham-OVX resilient+vehicle: 1.04 $\pm$ 0.09; Sham-OVX helpless+vehicle: 0.62 $\pm$ 0.05, n = 3 brains/group). Full-length blot is presented in Supplementary Figure S14. Data are represented as means $\pm$ SEM (One-way ANOVA Tukey’s post hoc test, *p < 0.05, **p < 0.01, ***p <0.001).


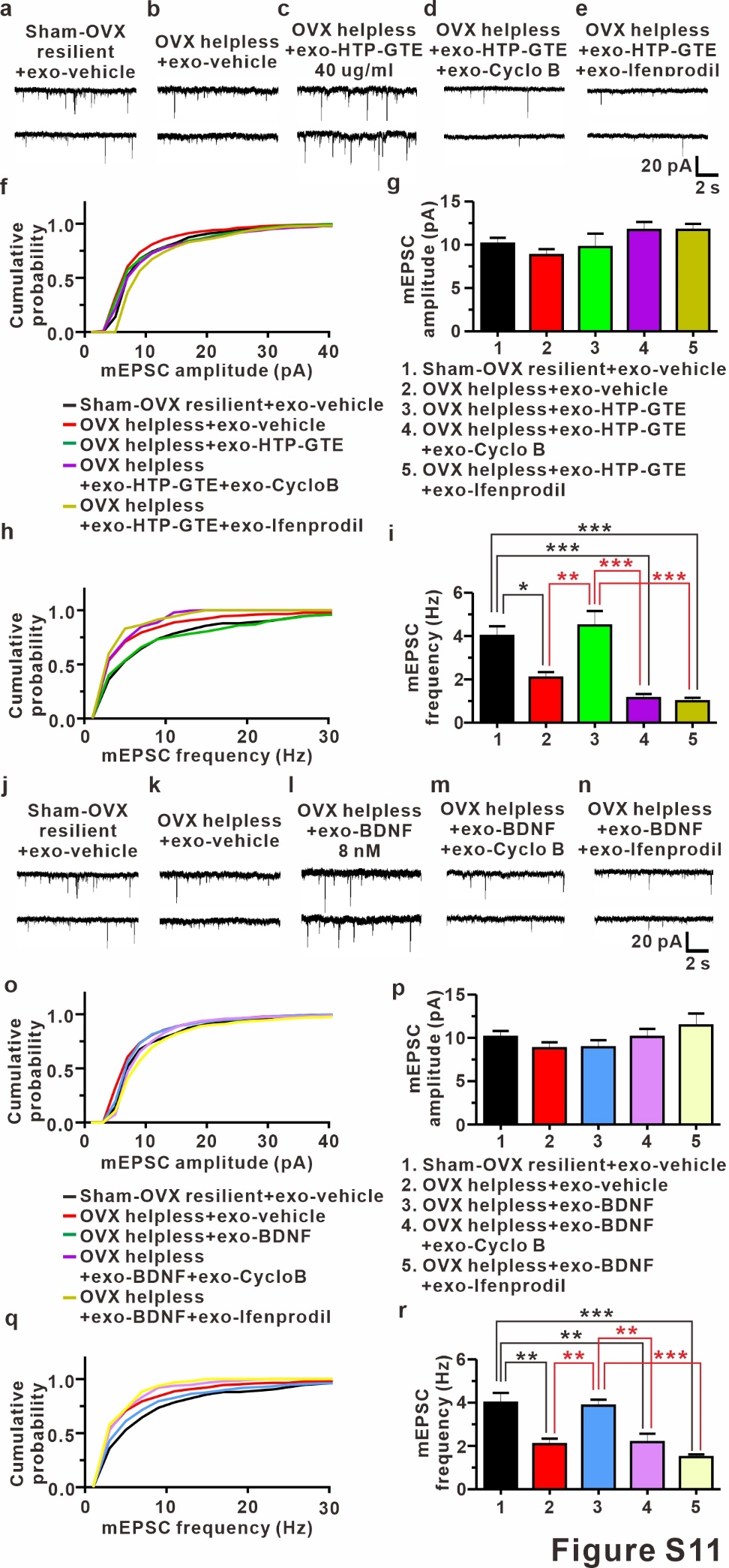


**Fig. S11. Regulation of BDNF-TrkB pathway underlies HTP-GTE-dependent amelioration of hippocampal synaptic impairment in helpless OVX rats.**

**a-e**, Effects of exogenous application of HTP-GTE were measured with spontaneous mEPSC in hippocampal slices. Sample traces showed miniature EPSC for Sham-OVX resilient+exo-vehicle, OVX helpless+exo-vehicle, OVX helpless+exo-HTP-GTE 40 μg/ml, and OVX helpless+exo-HTP-GTE+exo-Cyclo B, OVX helpless+exo-HTP-GTE+exo-Ifenprodil groups **f**, Cumulative probability plots of mEPSC amplitudes for each group (Sham-OVX resilient+exo-vehicle vs OVX helpless+exo-vehicle: p=0.9952; Sham-OVX resilient+exo-vehicle vs OVX helpless+exo HTP-GTE 40 μg/ml: p=0.9952; Sham-OVX resilient+exo-vehicle vs OVX helpless+exo-HTP-GTE+exo-Cyclo B: p=0.3027; Sham-OVX resilient+exo-vehicle vs OVX helpless+exo-HTP-GTE+exo-ifenprodil: p=0.3027, Kolmogorov-Smirnov two-sample test). **g**, The mean amplitude of mEPSC for all groups (Sham-OVX resilient+exo-vehicle: 10.12 $\pm$ 0.69, n = 6 slices/3 rat; OVX helpless+exo-vehicle: 8.81 $\pm$ 0.69, n = 6 slices/3 rat; OVX helpless+exo-HTP-GTE 40 μg/ml: 9.73 $\pm$ 1.55, n = 6 slices/3 rat; OVX helpless+exo-HTP-GTE+exo-Cyclo B: 11.71 $\pm$ 0.92, n = 6 slices/3 rat; OVX helpless+exo-HTP-GTE+exo-Ifenprodil: 11.72 $\pm$ 0.69, n = 6 slices/3 rat). **h**, Cumulative probability plots of mEPSC frequencies for all groups (Sham-OVX resilient+exo-vehicle vs OVX helpless+exo-vehicle: p<0.05; Sham-OVX resilient+exo-vehicle vs OVX helpless+exo HTP-GTE 40 μg/ml: p=0.7222; Sham-OVX resilient+exo-vehicle vs OVX helpless+exo-HTP-GTE+exo-Cyclo B: p<0.001; Sham-OVX resilient+exo-vehicle vs OVX helpless+exo-HTP-GTE+exo-ifenprodil: p<0.001, Kolmogorov-Smirnov two-sample test). **i**, The mean frequency of mEPSCs for all groups (Sham-OVX resilient+exo-vehicle: 4.01 $\pm$ 0.45, n = 6 slices/3 rat; OVX helpless+exo-vehicle: 2.08 $\pm$ 0.25, n = 6 slices/3 rat; OVX helpless+exo-HTP-GTE 40 μg/ml: 4.49 $\pm$ 0.67, n = 6 slices/3 rat; OVX helpless+exo-HTP-GTE+exo-Cyclo B: 1.13 $\pm$ 0.19, n = 7 slices/3 rat; OVX helpless+exo-HTP-GTE+exo-Ifenprodil: 0.99 $\pm$ 0.16, n = 6 slices/3 rat). **j-n**, Effects of exogenous application of HTP-GTE were measured with spontaneous mEPSC in hippocampal slices. Sample traces showed miniature EPSC for Sham-OVX resilient+exo-vehicle, OVX helpless+exo-vehicle, OVX helpless+exo-BDNF 8 nM, OVX helpless+exo-BDNF+exo-Cyclo B, and OVX helpless+exo-BDNF+exo-Ifenprodil groups. **o**, Cumulative probability plots of mEPSC amplitudes for all groups (Sham-OVX resilient+exo-vehicle vs OVX helpless+exo-vehicle: p=0.9952; Sham-OVX resilient+exo-vehicle vs OVX helpless+exo BDNF 8nM: p=0.7222; Sham-OVX resilient+exo-vehicle vs OVX helpless+exo-BDNF+exo-Cyclo B: p=0.7222; Sham-OVX resilient+exo-vehicle vs OVX helpless+exo-BDNF+exo-ifenprodil: p=0.1705, Kolmogorov-Smirnov two-sample test). **p**, The mean amplitude of mEPSCs for all groups (Sham-OVX resilient+exo-vehicle: 10.12 $\pm$ 0.69, n = 6 slices/3 rat; OVX helpless+exo-vehicle: 8.81 $\pm$ 0.69, n = 6 slices/3 rat; OVX helpless+exo-BDNF 8 nM: 8.92 $\pm$ 0.81, n = 6 slices/3 rat; OVX helpless+exo-BDNF+exo-Cyclo B: 10.12 $\pm$ 0.92, n = 6 slices/3 rat; OVX helpless+exo-BDNF+exo-Ifenprodil: 11.44 $\pm$ 1.38, n = 6 slices/3 rat). **q**, Cumulative probability plots of mEPSC frequencies for all groups (Sham-OVX resilient+exo-vehicle vs OVX helpless+exo-vehicle: p<0.01; Sham-OVX resilient+vehicle vs OVX helpless+exo BDNF 8nM: p=0.918; Sham-OVX resilient+exo-vehicle vs OVX helpless+exo-BDNF+exo-Cyclo B: p<0.001; Sham-OVX resilient+exo-vehicle vs OVX helpless+exo-vehicle+exo-BDNF+exo-ifenprodil: p<0.001, Kolmogorov-Smirnov two-sample test). **r**, The mean frequency of mEPSCs for all groups (Sham-OVX resilient+exo-vehicle: 4.01 $\pm$ 0.45, n = 6 slices/3 rat; OVX helpless+exo-vehicle: 2.08 $\pm$ 0.25, n = 6 slices/3 rat; OVX helpless+exo-BDNF 8 nM: 3.86 $\pm$ 0.28, n = 6 slices/3 rat; OVX helpless+exo-BDNF+exo-Cyclo B: 2.18 $\pm$ 0.39, n = 6 slices/3 rat; OVX helpless+exo-BDNF+exo-Ifenprodil: 1.49 $\pm$ 0.12, n = 6 slices/3 rat). Data are represented as means $\pm$ SEM (One-way ANOVA Tukey’s post hoc test, **p < 0.01, ***p <0.001).


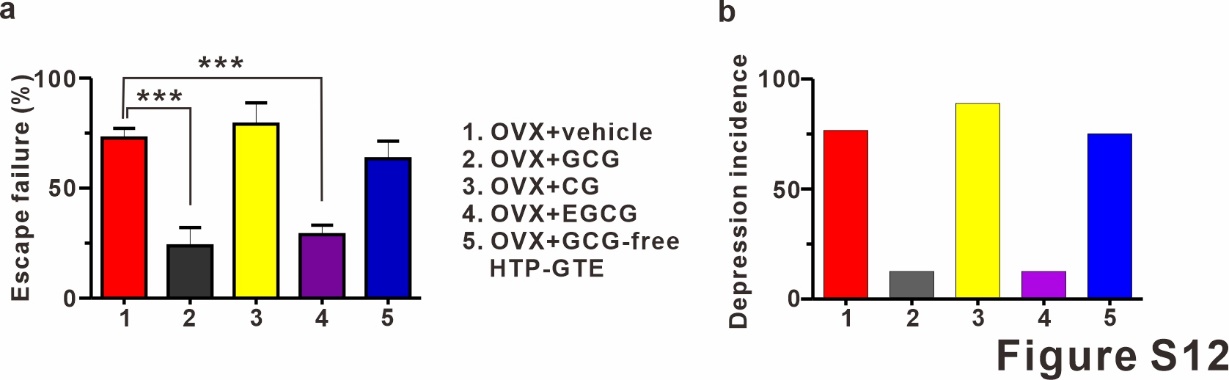


**Fig. S12. GCG, but not EGCG, plays a role as a major active component in HTP-GTE -induced improvements of synaptic and cognitive impairments in the helpless OVX rats.**

**a**, Escape failure for HTP-GTE component (GCG, CG, EGCG, and GCG-free HTP-GTE )-treated OVX rats (OVX+vehicle: 72.76 $\pm$ 4.43, n = 29; OVX + GCG: 23.81 $\pm$ 8.34, n = 7; OVX + CG: 79.17 $\pm$ 9.59, n = 8; OVX + EGCG: 28.89 $\pm$ 4.30, n = 9; OVX + GCG-free HTP-GTE : 63.33 $\pm$ 8.04, n = 11). **b**, Depression incidence was shown. Data are represented as mean $\pm$ SEM (One-way ANOVA Tukey’s *post hoc* test, ***p < 0.001).


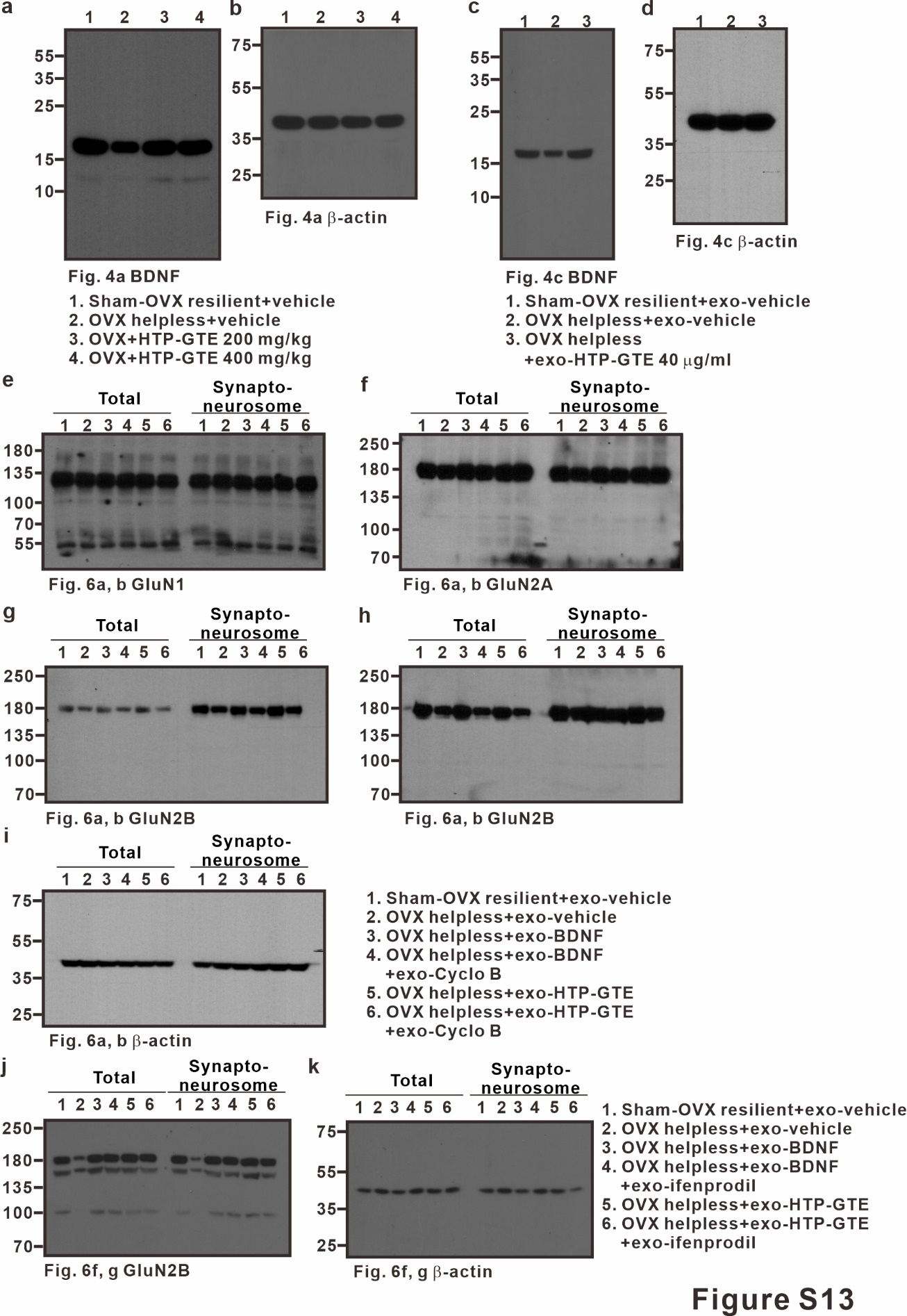


**Fig. S13. Full-length blot of main figures**

**a**, BDNF blot in fig.4a. **b**, β-actin blot in fig.4a. **c**, BDNF blot in fig.4c. **d**, β-actin blot in fig.4c. **e-i**, GluN1, GluN2A, GluN2B and β-actin blot in fig. 6a, b. **j-k**, GluN2B blot and β-actin blot in fig. 6f, g.


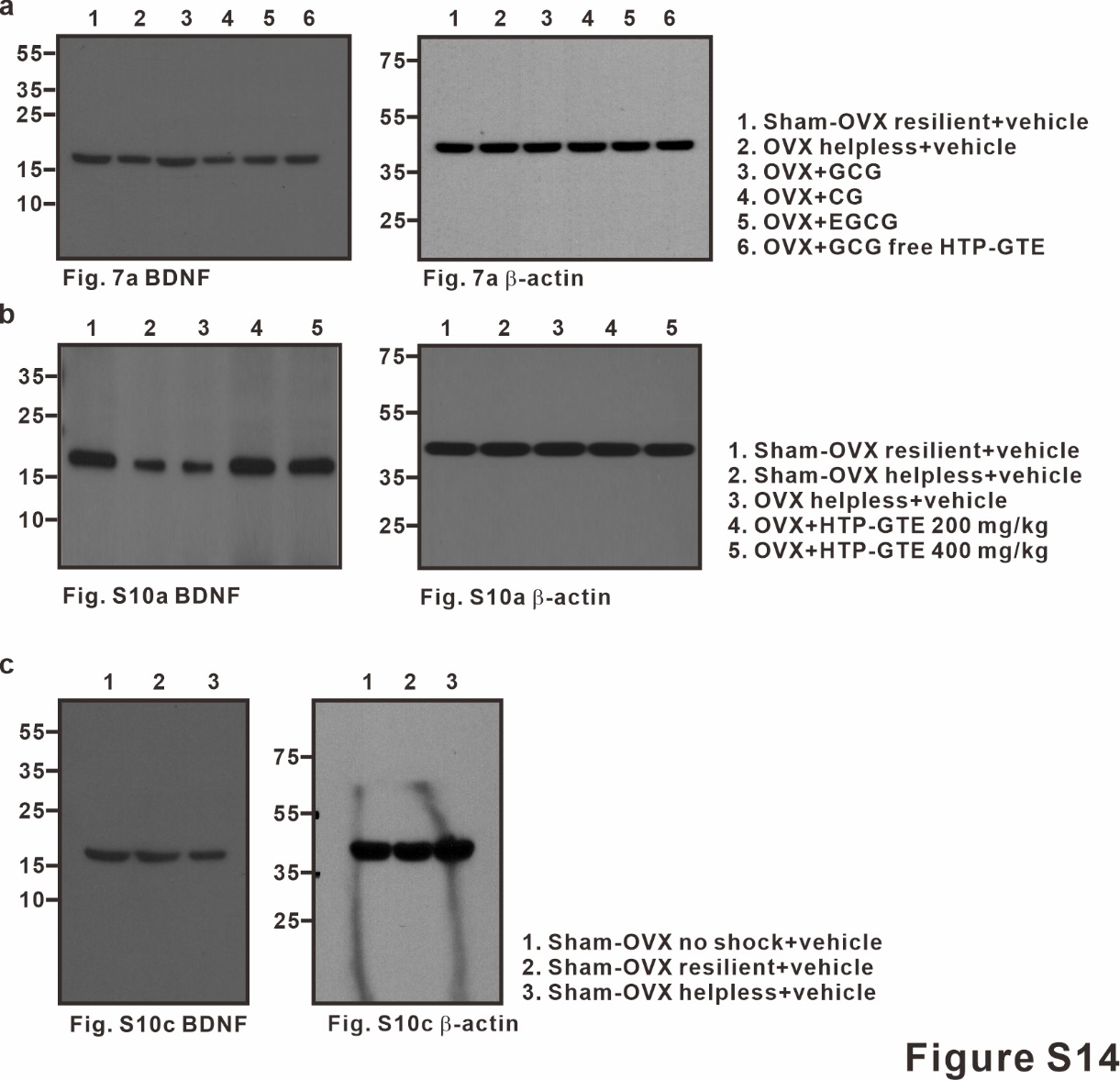


**Fig. S14. Full-length blot of main figures**

**a**, BDNF and β-actin blot in fig.7a. **b**, BDNF and β-actin blot in fig. S9a. **c**, BDNF and β-actin blot in fig. S9c.


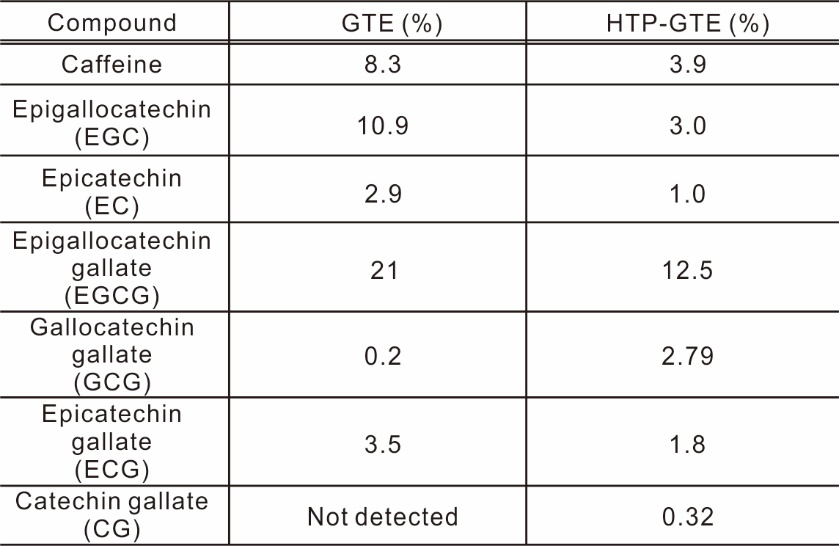


**Table S1. Composition of catechins and caffeine of GTE and HTP-GTE.**

EGC: Epigallocatechin; EC: Epicatechin; EGCG: Epigallocatechin gallate; GCG: Gallocatechin gallate; ECG: Epicatechin gallate; CG: Catechin gallate
